# Iron Sulfides Produced by Thermococcales: An Iron Detoxification Mechanism

**DOI:** 10.1111/1462-2920.70242

**Published:** 2026-01-22

**Authors:** T. Mariotte, R. Coudray, C. Toffano‐Nioche, F. Guyot, A. Gorlas

**Affiliations:** ^1^ Université Paris‐Saclay, CEA, CNRS Institute for Integrative Biology of the Cell (I2BC) Gif‐sur‐Yvette France; ^2^ Institut de Minéralogie, de Physique des Matériaux et de Cosmochimie (IMPMC) Sorbonne Université, Museum National d'Histoire Naturelle, CNRS Paris France

**Keywords:** biomineralization, detoxification, pyrite, Thermococcales

## Abstract

Thermococcales, sulfur‐reducing archaea inhabiting the hottest parts of hydrothermal vents, have evolved to thrive in environments rich in iron and sulfide species. In this study, using experimental analogues of sulfur‐rich hydrothermal chimneys, we confirm previous suggestions that the precipitation of iron sulfide minerals promoted by Thermococcales contributes to a population‐wide adaptation to reactive species induced by the presence of high levels of iron. In parallel with mineral phases identification, cellular metabolic activity was monitored during mineralization, revealing a mechanism in which a subpopulation of cells does not survive mineralization and becomes encrusted in pyrite, while the remaining living cells exhibit a gene expression profile focused on DNA repair and metal excess associated detoxification. Compared to abiotic conditions, Thermococcales induce a faster precipitation of dissolved iron, immobilising excess metal. Our results clarify the role of mineralizing cells in this survival mechanism, suggesting that this biomineralization process allows resilience to extreme chemical stress. Upon drastic levels of toxic dissolved iron, thanks to a population of mineralizing cells, the surviving Thermococcales are thus more likely to endure those still harsh environments. This complex mechanism is likely a key factor in the adaptation of microorganisms to the hottest environments of hydrothermal vents.

## Graphical abstract

Thermococcales, archaea from hydrothermal vents, promote iron sulfide precipitation, enabling survival in iron‐rich environments. Some cells become encrusted in pyrite and do not survive mineralization, while surviving cells activate metal detoxification genes. This biomineralization process allows the population to withstand toxic iron concentrations, supporting adaptation to extreme, high‐temperature hydrothermal conditions.

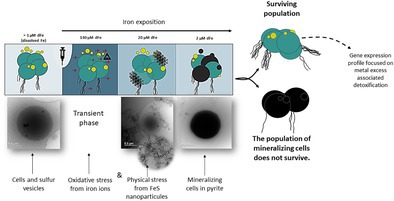

## 1 Introduction

Living organisms require metallic ions to perform essential functions. Cells utilize metals, particularly iron and copper for their electron transfer capabilities. Other metals such as zinc, cobalt and magnesium often act as electrophilic centres. Proteins structure also depends on metals, as domain folding is often organized around zinc or calcium (Merchant and Helmann 2012). Studies suggest that metal cofactors are present in at least 30% of proteins across most organisms (Waldron et al. 2009; Seravalli and Ragsdale 2010). However, excessive exposure to metals can cause cellular damage, including protein and lipid peroxidation, as well as DNA fragmentation (Nel et al. 2006). Metal‐induced dangers to cells include mismetallation of enzymes and regulators (Chandrangsu et al. 2017), as well as reactive oxygen species (ROS) production (Touati 2000). The resulting oxidative stress may in turn coincide with physical stress from nanoparticles, which can damage membrane integrity (Karlsson et al. 2013; Li et al. 2018). Iron, in particular, is known to induce ROS production through the Fenton reaction, and studies have uncovered mechanisms through which iron‐based nanoparticles damage cells (Tapley et al. 1999). Microorganisms address these stresses by restoring iron homeostasis and managing its resulting effects by limiting the Fenton reaction and repairing DNA. They balance acquisition, efflux, and storage of iron through pumps, ferritins, and siderophore regulation, while ROS are detoxified using a range of antioxidants.

Anoxic and microaerophilic environments are not exempt from oxidative stress. In hydrothermal vents, sulfide oxidation catalyzed by metals leads to hydroxyl radicals production (Tapley et al. 1999). Hydrothermal vents are characterized by steep physicochemical gradients. For instance, the concentration of dissolved iron (dFe) can vary widely, from micromolar to hundreds of millimolar, depending on the site and the position in the vent chimney (Von Damm 1995; Holden et al. 2012; Waeles et al. 2017; Yücel et al. 2021). Thermococcales, common inhabitants of the hottest parts of black smokers (Takai et al. 2001), are exposed to high concentrations of dFe. They have been shown to induce the biomineralization of greigite and pyrite nanocrystals (Gorlas et al. 2022). The term “biomineralization” (BM) is used here to describe a wide range of cell–mineral interactions, involving various biological functions, levels of control over mineral nucleation and growth. Gorlas et al. (2022) suggested that the BM of Thermococcales could function as a survival mechanism in metal‐rich hydrothermal environments following observations showing that mineralized (dead) cells could shield part of the population from transient flushes of toxic hydrothermal fluids and then provide nutrients to surviving cells recovering from a quiescent low‐metabolic state (Gorlas et al. 2022).

With a view to testing this hypothesis, we demonstrate in this study that Thermococcales induce faster precipitation of dFe compared to abiotic conditions. While a part of the population is fully encrusted in pyrite, the remaining living cells exhibit a gene expression profile oriented toward DNA repair and heavy‐metal detoxification. Despite this, the surviving population maintains a similar level of function and growth efficiency in an iron‐rich medium as cells grown under low optimal iron concentrations. Our study aims to elucidate the role of BM in this adaptation to highly mineralizing environments, and to further elucidate its molecular mechanisms. We also clarify the role of mineralizing cells in this proposed adaptive mechanism, suggesting that the BM process allows Thermococcales to resist, at the population level, higher concentrations of iron than those permitted by conventional detoxification mechanisms, through enhanced metal immobilization. As a result of this sacrificial metal immobilization, the remaining live cells are better equipped to manage residual dFe concentrations through gene‐encoded iron detoxification mechanisms, increasing their chances of survival.

## 2 Materials and Methods

### 2.1 Culture Conditions

*Thermococcus kodakarensis* KOD1 (JCM 12380) was cultivated at 85°C in modified Ravot medium, prepared as previously described (Gorlas et al. 2022) containing per litre of distilled water: 1 g NH_4_Cl, 0.2 g MgCl_2_.6H_2_O, 0.1 g CaCl_2_.2H_2_O, 0.1 g KCl, 0.83 g CH_3_COONa.2H_2_O, 20 g NaCl, 1 g yeast extract, 1 g tryptone, 3.45 g PIPES buffer (piperazine‐N,N9‐bis (2‐ethanesulphonic acid)) adjusted to pH 7, and autoclaved. After autoclaving, the following sterile solutions were added aseptically: 2.5 mL of 6% (w/v) K_2_HPO_4_ solution and 2.5 mL of 6% (w/v) KH_2_PO_4_ solution. The medium was then dispensed (50 mL) into 100 mL sterile flasks and supplemented with 1 g (w/v) elemental sulfur L^−1^. Anaerobiosis was obtained by applying a vacuum to the medium and saturating it with dinitrogen. Finally, a sterile solution of Na_2_S.9H_2_O [final concentration 0.05% (w/v)] was added to reduce the medium.

### 2.2 Biomineralization Experiments

*T. kodakarensis* cells were grown in modified Ravot medium supplemented with elemental sulfur (1 g/L). When cells were close to stationary phase, ferrous sulfate (FeSO_4_) or ferrous chloride (FeCl_2_) solutions were added to the culture and in cell‐free control medium at final concentrations of 5.0, 1.0, or 0.1 mM. Cultures were incubated at 85°C for a maximum of 30 days, with sampling at different times throughout the experiment. Additional experiments were conducted at lower temperatures, namely 25°C and 45°C. Some experiments were also performed with cells in the late exponential growth phase, fixed with glutaraldehyde solution (cf. 2.5%).

### 2.3 Intracellular ATP Concentration

Adenosine triphosphate (ATP) is consumed in the conversion of luciferin to oxyluciferin catalysed by the luciferase enzyme. This reaction leads to the emission of luminescence that can be measured by a luminometer. Since ATP in the sample is the limiting reagent, the bioluminescence is proportional to the ATP concentration. Microbial ATP kit HS (BioThema) was used in these experiments and bioluminescence was measured with a Kikkoman luminometer, as previously described in Gorlas et al. (2022).

### 2.4 Cytometry Analyses

Samples were fixed with glutaraldehyde (2.5% final concentration) then frozen with liquid nitrogen for storage at −80°C. Samples were marked with SYBR Green (1× final concentration from a 10,000× Sigma‐Aldrich solution) and filtered with CellTrics (Sysmex) 10 μM cut‐off filters to prevent clogging. Cell concentrations were measured using the Cytoflex bench‐top cytometer (Beckman‐Coulter). Cytometry acquisition and analyses were performed using CytExpert 2.5 software (Beckman‐Coulter).

### 2.5 Confocal Microscopy

Samples for confocal fluorescence microscopy were prepared on agar pads (low‐melting 1.5% agar) on glass slides. Samples were marked with either SYBR Green (1× final concentration) or an Invitrogen Live‐dead kit mix of propidium iodide (20 mM initial, 0.2 mM final) and Syto9 (3.34 mM initial, 0.03 mM final) or Sigma‐Aldrich Nile red (2.5 μg/mL final). Samples were observed using the Leica SP8 Laser scanning confocal microscope and the Leica Application Suite X (LAS X) 3.5.7.

### 2.6 RNA Extraction and Transcriptomic Analyses

Cell culture pellets were obtained by centrifugation at 5000 *g* for 30 min at 10°C. Cellular total RNAs were extracted with a NucleoSpin RNA set for NucleoZOL (Macherey‐Nagel). The kit was used to precipitate contaminants such as DNA, proteins, and polysaccharides. Supernatants, containing RNAs, were then eluted on NucleoSpin RNA Column, made of silica, resulting in column‐bound RNAs. Samples were then washed and RNAs were eluted in 50 μL of water. Samples were loaded on a 1.8% agarose gel to verify their quality. Amplification of DNA corresponding to 16S rRNA was performed by PCR to check for possible DNA contamination. A control containing *Thermococcus kodakarensis* KOD1 DNA was used. The 16S rRNA gene sequence was amplified by PCR using the forward and reverse primers 4F specific for Archaea (5′‐TCCGGTTGATCCTGCCGG‐3′) and 1492R universal (5′‐GGTTACCTTGTTACGACTT‐3′). The reaction was performed in a volume of 50 μL containing 50 ng template, 10 mM of each primer, 10 mM dNTPs, 25 mM MgCl_2_, 1× buffer (Promega), and 1 U polymerase. RNA concentration of the samples was measured using a DS‐11 Spectrophotometer (Denovix) while RNA quality was evaluated using a Qubit 4 fluorometer (Thermo Fischer) by IQ RNA dosage.

Total RNA extracts (with RIN > 8.5) were sent to the I2BC sequencing platform, where library preparation and sequencing (RNA‐seq) were performed. Ribosomal RNAs were depleted using the Ribo‐Zero kit, followed by reverse transcription to generate complementary DNA (cDNA) using the ScriptSeq protocol. Sequencing was carried out using the Illumina technology on a NextSeq Instrument using a NextSeq 2000 P2 kit (100 cycles, single‐read), with a total of up to 300 million reads guaranteed. The raw sequencing data were demultiplexed using bcl‐convert (v3.9.3), adapters were trimmed with Cutadapt (v3.2), and quality control was assessed using FastQC (v0.11.5). Across the 12 samples (covering 4 conditions: 3 samples T0hFe corresponding to cells cultures after iron induction, 3 samples T72hFe corresponding to cells cultures after iron induction followed by 72 h of mineralization, and the two control conditions without iron addition: 3 samples T0h and 3 samples T72h) sequencing generated between ~25 and 40 million mapped reads per library, with highly consistent read distributions among biological replicates. A total of 2289 annotated features were detected in the count matrix, and only one feature (0.04%) showed zero counts across all samples, indicating an excellent transcriptome coverage. Downstream analysis was conducted using a snakemake bioinformatics workflow (Kreis et al. 2024; Koster and Rahmann 2018). Reads were aligned to the *Thermococcus kodakarensis* KOD1 reference genome (GCF_000009965.1_ASM996v1 downloaded from the NCBI) using Bowtie 2 (v2.4.1) (Langmead and Salzberg 2012) and mapped reads were selected with samtools (v1.13) (Li et al. 2009). Gene expression quantification was performed with FeatureCounts (from the subread package, V2.0.1) (Liao et al. 2014), and differential gene expression analysis was carried out using DESeq2 (v1.40.2) (Love et al. 2014). Genes (excluding rRNA and tRNA ones) showing a minimum fold change of 2 and a *p*‐value < 0.001 were considered significantly differentially expressed.

### 2.7 Iron Titration

Samples supernatants were obtained by centrifugation at 11,000 g for 5 min. The dosages were performed with colorimetric methods. A solution at 10 mM was first obtained by dissolving 0.0162 g of FeCl_3_ in 10 mL of H_2_O. Fe^3+^ standards are concentrated at 250, 500, 750, 1000 and 10,000 μM. The standards and the samples were acidified with HCl 6N. Solutions of hydroxylamine in HCl 6N were added to reduce Fe^3+^. Samples and standards were incubated 1 h 30 at 60°C. Ferrozine and ammonia buffer solution (pH 10) were then added. The absorbance at 562 nm was measured with a Cary 50 spectrometer (Varian).

### 2.8 TEM–SEM–Cryo‐TEM

Mineral phases and cell‐minerals interactions were observed by using scanning electron microscopy (SEM), transmission electron microscopy (TEM), cryo transmission electron microscopy (cryo‐TEM) and light microscopy.

For TEM observations, 10 μL droplets of sample were adsorbed onto a carbon‐coated nickel grid. The excess liquid was then removed, and the grid was washed with sterile water. Samples were observed at 80 kV or 120 kV in JEOL TEM equipped with LAB_6_ gun. FIB sections (see below) were observed using a JEOL 2100F Field Emission Gun (FEG) TEM.

For SEM observations, 2 mL of sample was filtered on 0.22 μm membrane, which were fixed on aluminium plots with carbon tape. Samples were observed by SEM (Zeiss Ultra55). Carbon‐coated nickel grids were also observed by SEM using a specific sample holder.

Cryo‐TEM samples were prepared as described in Gorlas et al. (2022), and observed under cryogenic conditions in a JEOL 2100 LaB6 TEM, operating at an accelerating voltage of 200 kV under a nominal magnification of 10,000. Images were captured under low dose conditions using an ultra‐scan 1000 camera (Gatan, 2 k × 2 k pixels CCD).

Elemental analysis was performed through energy‐dispersive X‐ray spectroscopy (EDXS). For TEM, a Si(Li) detector with ultrathin polymer window was used (JEOL, resolution: 140 eV/channel). For SEM, EDXS was carried out using BRUKER silicon drift technology with polymer window (resolution: 123 eV/channel at 100kcounts/s). The EDXS analyses were combined with TEM, SEM and Cryo‐EM techniques.

### 2.9 Focused Ion Beam

Focused ion beam (FIB) foils (20 μm x 5 μm x 100 nm) were extracted from pyrite spherules using a FEI Strata DB 235 (IEMN, Lille, France). Milling at low gallium ion currents allowed minimising common artefacts including local gallium implantation, mixing of components, redeposition of the sputtered material on the sample surface, and significant changes in the speciation of carbon‐based polymers.

### 2.10 Powder X‐Ray Diffraction

X‐Ray Diffraction (XRD) samples were prepared from 50 mL of live culture centrifuged at 5000 g for 30 min. Supernatants were preserved at 4°C for colorimetric iron dosages while pellets were washed with a first cycle of water to remove excess salt and elemental sulfur; then a washing cycle was performed with ethanol. Samples were then dried in anaerobic conditions (in N_2_ atmosphere, in Jacomex glove box). Diffraction patterns were acquired using a XPert Pro Panalytical diffractometer with Co Kα radiations. The settings used for acquisition were continuous scan mode with a 0.0170° 2*θ* step from 5° to 90° 2*θ* over 3 h per sample. Data analysis was performed with Highscore Plus software, using sulfur pattern (Coppens et al. 1977) and pyrite pattern (Finklea et al. 1976).

## 3 Results

### 3.1 Formation of FeS_2_ Pyrite Spherules in Presence of Thermococcales Cells

**FIGURE 1:**
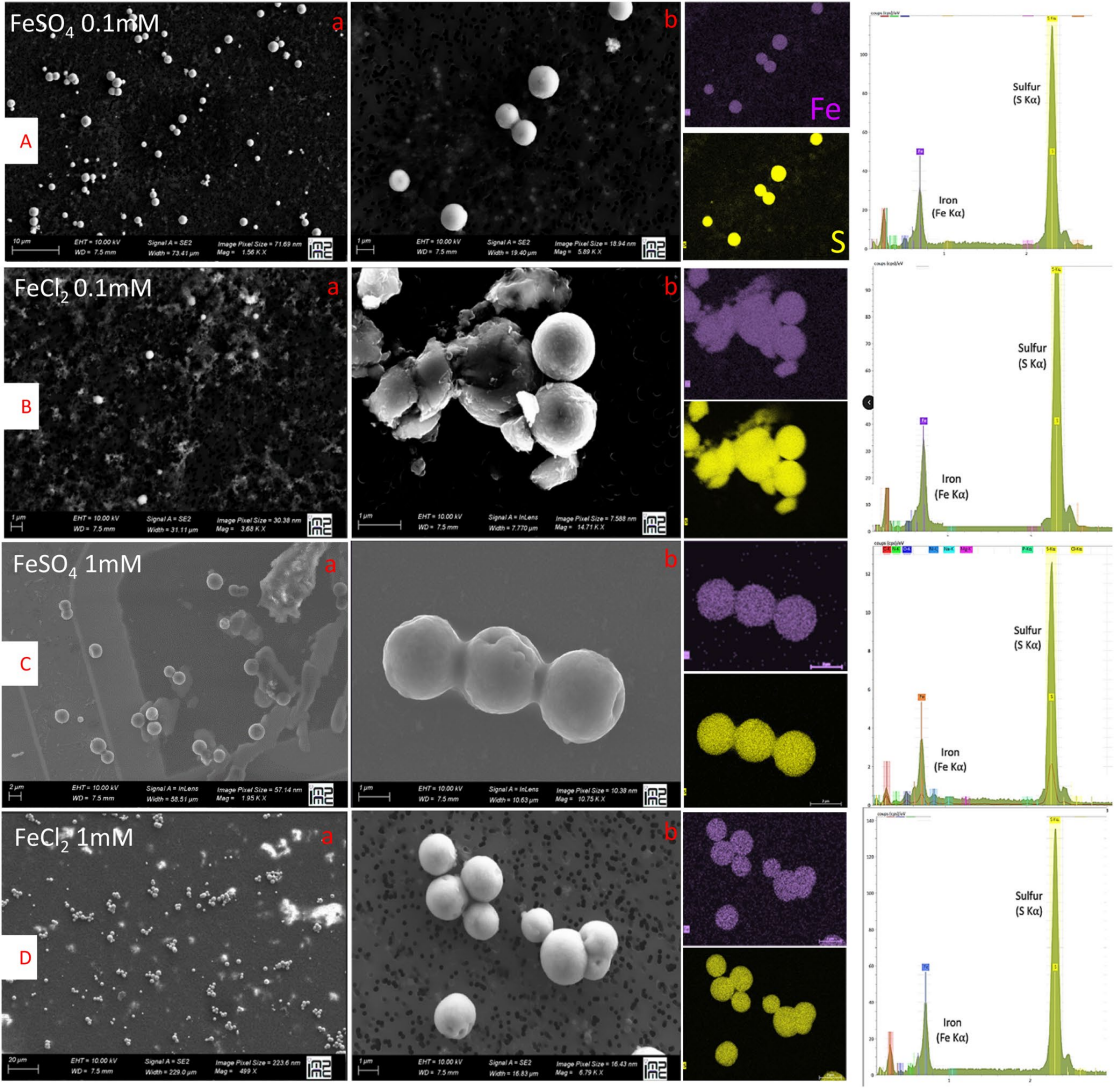
Iron‐sulfide spherules production after 20 days of incubation of *T. kodakarensis* in sulfur medium containing FeSO_4_ (A, C) or FeCl_2_ (B, D) at 0.1 mM (A, B) or 1 mM (C, D). Scanning back scattered electron microscopy image of mineralized samples, associated EDXS (energy dispersive X‐ray diffraction) maps and spectra.

**FIGURE 2:**
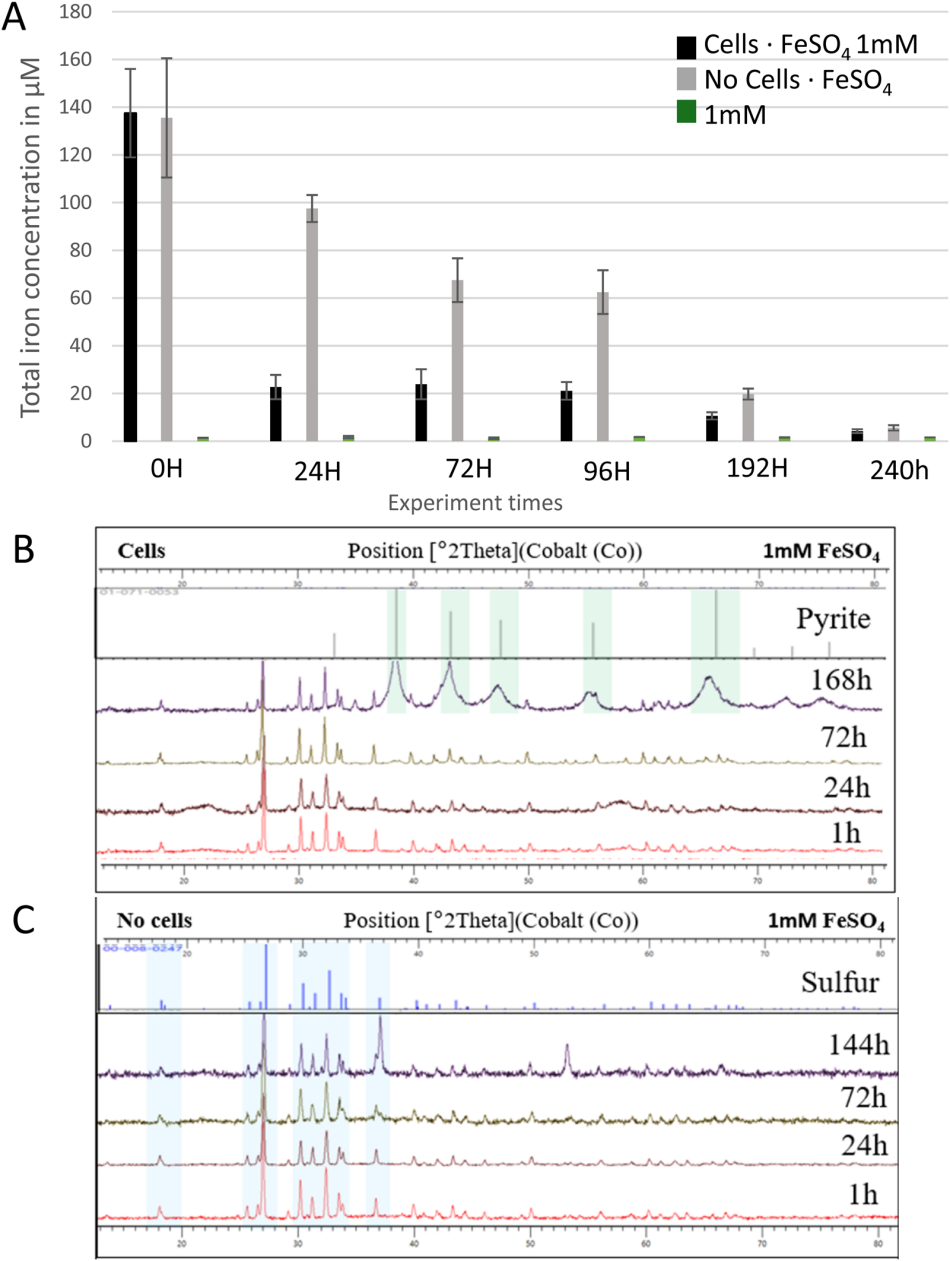
Concentration of total dissolved iron (in μM) in medium at different times after adding 1 mM FeSO_4_ at 0H, in presence of cells (black markers) or in absence (grey markers) (A). A culture control is conducted in absence of FeSO_4_ addition (green markers). X‐ray diffraction (XRD) patterns of induced culture samples in the presence (B) and absence (C) of cells at times counted from the iron induction. Over the superposed diffraction patterns of the samples are the reference patterns of S_8_ sulfur and pyrite. Major peaks are highlighted to indicate the presence of each phase in the samples. Samples without highlighted peaks show no significant signal for the identification of the phase of interest.

Upon addition of iron (FeCl_2_ or FeSO_4_) at different concentrations (0.1 and 1 mM), spherules of pyrite appear after a few 10 h (Figure 1A–D) only in the presence of cells. An analysis of selected area electron diffraction (SAED) patterns of the spherule interior was conducted on ultrathin sections prepared by FIB studied by TEM. SAED patterns characteristic of FeS_2_ pyrite were obtained (Figure S1) without any evidence for other phases in the spherules. This identification is consistent with X‐ray diffraction (XRD) results, which also revealed the presence of pyrite (Figure 2B). As previously reported under mineralization conditions with FeSO_4_ at 5 mM, Thermococcales cells are mineralized, leading to the production of pyrite spherules (Gorlas et al. 2022; Truong et al. 2023). The pyrite spherules produced under the different conditions tested in this study display similar characteristics in terms of size (ranging from 400 nm to 2 μm), shape, and smooth surface texture. Under conditions of low iron concentration (0.1 mM FeSO_4_), pyrite spherules are still observed (Figure 1Aa,Ba), organised into clusters of two to three mineralized cells found isolated in the sample (Figure 1Aa,Ab,Ba,Bb). In contrast, clusters of large numbers of aggregated spherules are observed under conditions with FeSO_4_ at 1 mM (Figure 1Ca,Da). These clusters include several mineralized cells ranging between three (Figure 1Cb,Db) to seven (Figure 1Da). Such aggregation is even more evident when cells were incubated with FeCl_2_ instead of FeSO_4_ at the same concentrations. Samples were regularly collected at different time points up to 96 h of mineralization and then at 20 days of mineralization to determine the first pyrite spherules produced in each condition and their evolution. Interestingly, while the first pyrite spherules are observed after 72 h of mineralization with FeSO_4_ at 1 mM (Figure S2A), they are clearly detected earlier with 1 mM FeCl_2_, specifically after 24 h of mineralization (Figure S2B). Under both 0.1 mM FeSO_4_ and FeCl_2_ conditions, the first occurrences of pyrite spherules are distinctly identified after 20 days of mineralization only (Figure S2C,D). For each iron condition, control experiments without cells were conducted and no pyrite spherules were observed at any time and even after 20 days of mineralization.

To identify the essential factors of pyrite spherule formation, we tested additional conditions by conducting similar experiments over 20 days at 25°C and 45°C, in addition to the usual condition of 85°C (Figure S3). Additionally, we performed biomineralization experiments on cells fixed with glutaraldehyde, followed by washing steps, over 20 days (Figure S3). This fixation process led to cell death and resulted in the leakage of some intracellular content during the washing steps, while preserving the external cellular structure (Figure S3A). To evaluate whether the washing procedure could remove any biofilm produced by the cells, we also conducted parallel experiments on glutaraldehyde‐fixed cells without any washing steps. At the lower temperatures of 25°C and 45°C, pyrite spherules were still observed, but in smaller amounts than at 85°C (Figure S3B–D). The amount of spherule production was found to be temperature‐dependent, with the ratio of observable pyrite spherules at 25°C (Figure S3B) being approximately 1:10 compared to 45°C (Figure S3C), and 1:100 compared to 85°C (Figure S3D). In contrast, the glutaraldehyde fixation (with and without washing steps) did not result in any pyrite spherule production (Figure S3A).

*Thermococcus kodakarensis* cells are thus shown to be able to promote the formation of FeS_2_ pyrite, in the form of smooth spherules, in diverse laboratory settings, using different iron sources (FeSO_4_ or FeCl_2_), at varying initial concentrations of dissolved iron (from 0.1 to 1 mM and 5 mM as shown in previous studies), and at different temperatures (ranging from 25°C to 85°C). However, cells fixed with glutaraldehyde are not capable of producing pyrite.

### 3.2 Effect of Thermococcales Cells on Iron Precipitation

Total dissolved iron concentrations were measured in the different experiments both in presence and absence of cells (Figure 2). Under conditions without Fe^2+^ in medium, the total dissolved iron remained close to 1 μM over the 240 h duration of the experiment (Figure 2A, green marker). Upon addition of a 1 mM FeSO_4_ solution, the concentration of dissolved iron immediately dropped from 1 mM to values ranging between 110 and 160 μM, both in the presence and absence of cells (Figure 2A, black and grey markers, at the beginning of the experiment (0H)). Throughout the experiments, the concentration of dissolved iron decreased in both conditions, whether in presence or absence of cells. However, in presence of cells, it drastically decreased after 24 h of iron induction, reaching about 20 μM, whilse in the absence of cells, this decrease was less pronounced, maintaining values of up to 100 μM (Figure 2A, black and grey markers, at 24H). More precisely, in absence of cells, dissolved iron concentrations slightly decreased from 100 to 70 μM over at least 96 h whereas they were maintained at about 20 μM in presence of cells. Then, a decrease in dissolved iron concentrations was observed in both conditions (with or without cells) at 192 h of experiment, and concentrations dropped to about 20 μM in the absence of cells and to 10 μM in the presence of cells (Figure 2A, black and grey markers, at 192H). After 10 days of mineralization, dissolved iron concentrations were very low in both conditions (about 5 μM). Therefore, the presence of cells allowed faster iron precipitation than in abiotic controls and lower iron concentrations to be reached more than 100 h earlier than observed in abiotic conditions.

The addition at time 0H of Fe^2+^ to the culture medium of Thermococcales rapidly generated the formation of black precipitates, clearly visible in the culture flask (Figure S4A) and under optical microscopy (Figure S4B) in line with the immediate decrease in dissolved iron from concentrations of 1 to 110–160 μM. Numerous precipitates, forming an amorphous or poorly crystalline mineral matrix mainly composed of FeS, were consistently observed by transmission electron microscopy in those samples (Figure S5). Then at later stages in presence of cells, pyrite spherules appeared and became more and more abundant (see previous section). Figure S5 illustrates these findings with a chronological series of transmission electron microscope images showing the progression of iron precipitation in the presence of cells (Figure S5A) under 1 mM FeSO_4_ condition, as well as the suggestive mineralization of cells into pyrite spherules (see Section 4). The FeS matrix envelops both intact and lysing cells and images are suggestive of a penetration of the cellular membrane by this mineral nanomaterial (Figure S2). Over the course of the experiments in presence of cells, the density of the mineral matrix decreases, becoming almost non‐existent after 100 h of mineralization. Meanwhile, electron‐dense spherules begin to appear (Figure S5D–F). They are first observed in few numbers 72 h after induction and identified as pyrite (Figures S1 and S2A). Most of them become progressively more electron‐dense and increase in number (Figure S5D–E). After 144 h of mineralization, the pyrite spherules tend to gather, forming larger groups as the experiment continues. At 192 h, clusters of more than 20 spherules are often observed (Figure S5F). In controls without cells, no spherules are observed and the amorphous/poorly crystallised iron sulfide matrix remains dominant.

These observations are consistent with powder X‐ray diffraction's patterns of iron‐induced culture and control samples from 1 to 168 h after induction (Figure 2B,C). In all conditions, sulfur (S_8_) constitutes the major crystalline phase in the samples. In the abiotic control, sulfur is the only detectable crystalline phase, whereas in the presence of cells, pyrite remains undetected until 168 h, at which point its signal becomes strong.

### 3.3 Persistence of a Living Cellular Population Under 1 mM FeSO_4_ Condition

The 1 mM FeSO_4_ condition allows for the maintenance of a living cellular population throughout the mineralization experiment. Here, we monitored the persistence and viability of this non‐mineralizing cellular population. We used several techniques, including flow cytometry and ATPmetry, to respectively determine total cell numbers and cellular metabolic activity. Living and dead cells were observed under a confocal microscope using a live‐dead kit. Non‐mineralized cells were examined in detail using cryo‐electron microscopy. Additionally, a control condition corresponding to growing cells in the absence of iron was conducted.

**FIGURE 3:**
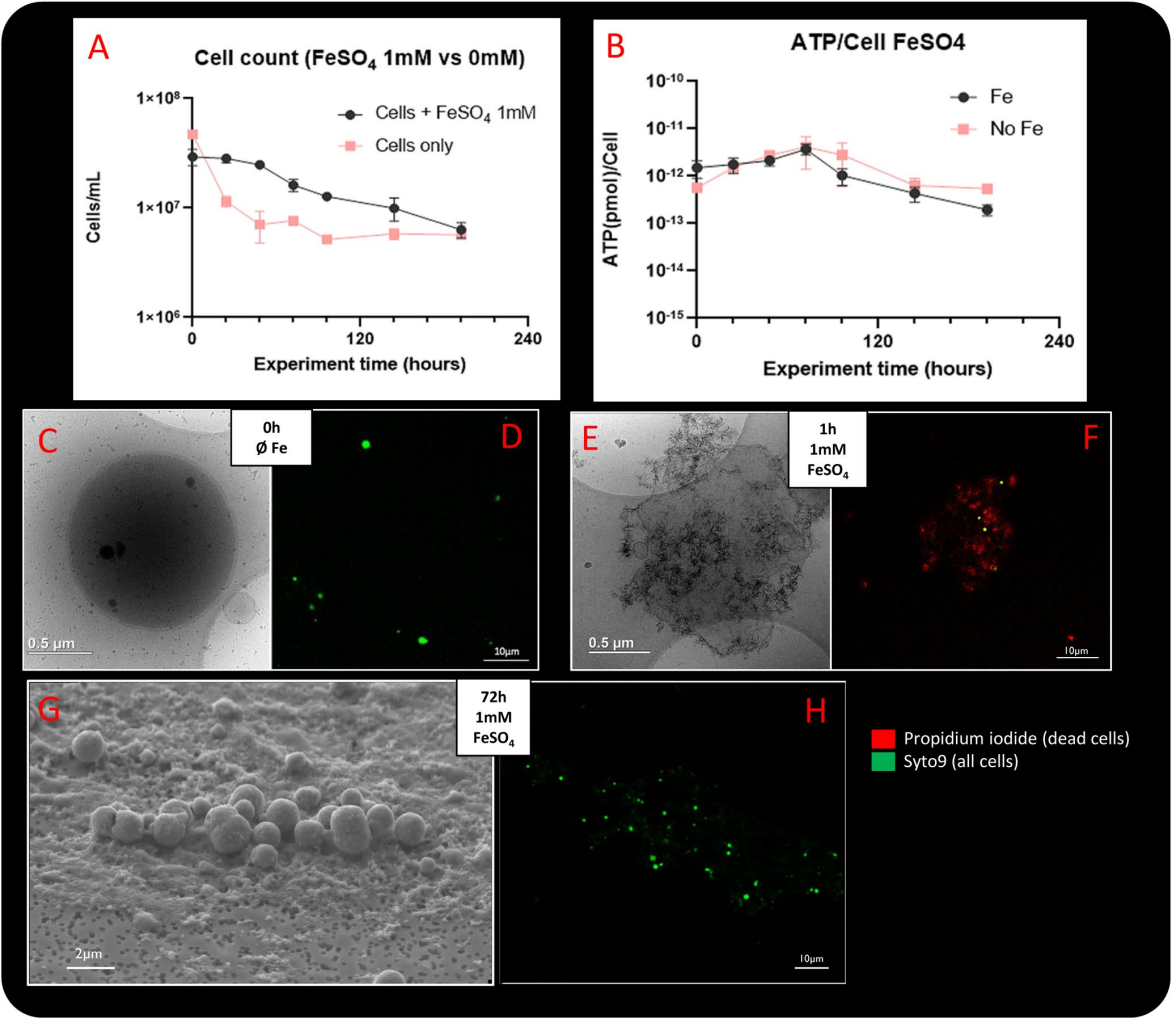
Cell count (A) and cellular ATP concentration (B) over BM experiment in *Thermococcus kodakarensis* culture with and without iron induction (A); SEM (G) and Cryo‐EM (C,E). Images of TK cells before (C), 1 h (E) and 72 h (G) after iron induction and (D, F, H) the corresponding fluorescence‐microscopy image. Dead cells marked with red fluorophore (IP) and live cells marked in green with Syto‐9.

Cell counts and ATPmetry were carried out to investigate the impact of a 1 mM concentration of FeSO_4_ on cells and were followed for 192 h after iron addition. In the control condition, the cell count decreases by about half a logarithmic unit within the first 24 h of monitoring, from 5 × 10^7^ to 1 × 10^7^ cells/mL, and stabilises thereafter at 9 × 10^6^ cells/mL (Figure 3A, pink line). Cultures with 1 mM FeSO_4_ exhibit a cellular concentration very similar to that of the iron‐free control condition, both in the initial and final measurements (Figure 3A, black line). While both conditions show a similar population decrease, this decline occurs at a notably slower rate after iron addition (Figure 3A). Importantly, flow cytometry counts include both live and dead cells, providing a total cellular concentration.

Additionally, ATP per cell concentration did not significantly differ between the two conditions (i.e., with and without iron addition, Figure 3B), with ATP concentrations starting at around 10^−12^ mmol/cell during the first 72 h of experiment and gradually decreasing to approach 10^−13^ mmol/cell at 192 h. ATP levels were normalized to the total cell number (live and dead) as determined by flow cytometry.

In parallel, live–dead experiments under a confocal microscope were performed to assess cells' viability, complementing the cell count and ATP concentration monitoring. Cells were then observed by scanning and transmission electron microscopies before iron addition, 1 and 72 h after (Figure 3C–H). In the control condition (without iron induction, Figure 3D), several scattered green dots, observable as foci of SYTO 9 signal, corresponding to live cells, are visible. Cryo‐electron microscopy (Figure 3C) shows intact cells with preserved membranes and S‐layers. No difference is observed after 72 h in this control condition.

In contrast, samples observed 1 h after iron induction consistently exhibit red clouds of propidium iodide signal containing small red dots, characteristic of dead cells. However, the same areas still show several living cells (represented by green dots). Over time, the proportion of dead cells decreases and living cells are again dominant within the mineralized environment, particularly after 72 h of induction.

Thus, confocal observations reveal a transient loss of viability shortly after iron addition, followed by a recovery phase during which live cells persist in the presence of minerals. This contrasts with the control condition (without iron induction), where the majority of the population remains viable throughout the experiment.

Cells are mostly co‐localized with amorphous iron–sulfur matrix, which emit a faint autofluorescence signal (Figure 3F). The colocalization of cells and mineral matrix is corroborated by electron microscopy. In cryo‐electron microscopy, damaged cells are observed (Figures 3E and S6). They display a deformed structure with a disrupted membrane lacking the S‐layer. Numerous mineral precipitates resembling needles are present close to cell surfaces and few vesicles protruding are observed. Fewer damaged cells are observed than in the case of higher concentrations of iron and the mineral matrix is only visible in a few areas of the observed sample (Figure S7). Some recurring core‐shell structures are observed, probably composed of a sulfur vesicle surrounded by FeS needles (Figure S8). After 72 h of mineralization, cells are still detected within the mineral matrix, but no dead cells are observed. Living cells are found close to the mineralized cells in pyrite (Figure 3H).

In the 1 mM FeSO_4_ conditions, although several dead and damaged cells are observed within the mineral matrix 1 h after iron addition, no significant differences in total cell count or ATP concentration are measured compared to the iron‐free control. However, it is important to note that these measurements include both live and dead cells, as total numbers were assessed by flow cytometry and ATP levels were normalised to total cell counts. Therefore, it cannot be excluded that a portion of the population was non‐viable, and that the observed ATP concentration reflects a higher metabolic activity in the remaining living cells. Nonetheless, no massive cell lysis is observed, as live‐dead staining under confocal microscopy clearly shows the presence of numerous living cells within the mineral matrix. After 72 h of induction, only living cells are detected, suggesting a recovery of the population and the persistence of a subpopulation that remains metabolically active despite mineralization.

### 3.4 Genome‐Wide Transcriptional Analyses After 72 h of Iron Induction

**FIGURE 4:**
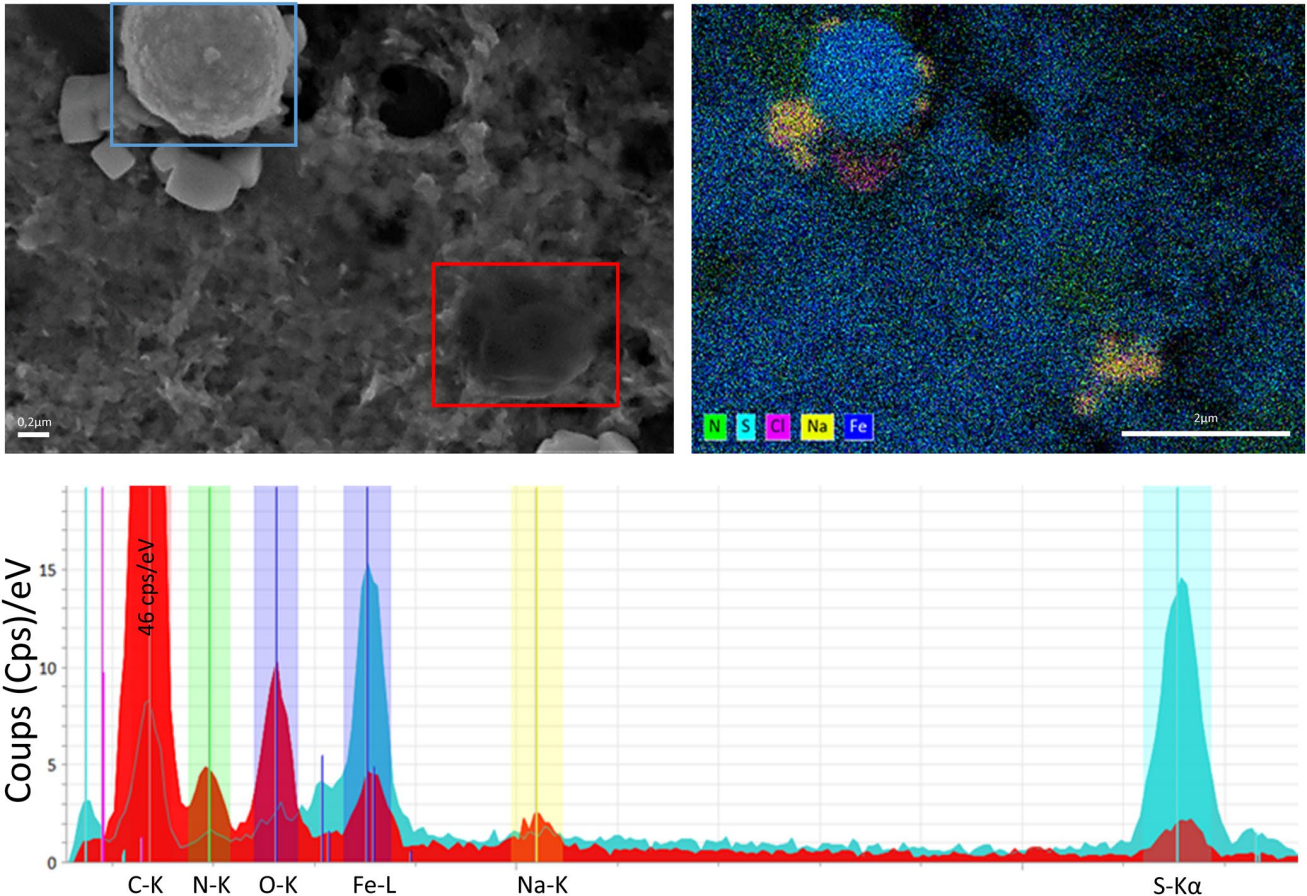
Scanning back scattered electron microscopy image of two *T. kodakarensis* cells, one mineralized (highlighted by a blue square) and one unmineralized (highlighted by the red square) (top left), along the EDX map overlay of nitrogen, sulfur, chloride, sodium and iron (top right) and the superposed EDX spectra of each cell (bottom). The EDX map allows the distinction of the iron–sulfur spherule and the organic nitrogen‐rich cell, both surrounded by sodium chloride crystals. The spectra confirm the respective mineral and organic content of the cells, with higher carbon, nitrogen and oxygen content of the unmineralized cell, as opposed to the higher iron and sulfur content of the mineralized cell.

To determine how iron influences gene expression profiles, transcriptomic analyses were conducted on cell cultures after iron induction, both immediately after (5 min corresponding to T0) and after 72 h of mineralization (corresponding to T72), as well as on control condition (cultures without iron addition) for comparative purposes. The decision to conduct transcriptomic analyses at the 72 h time point of mineralization is based on two key observations made at this time: pyrite spherules are observed while numerous living cells are still present (Figure 4).

Overall, this 72 h time point thus serves for examining the interplay between biomineralization and cellular responses. For each of the four conditions (i.e., T0 and T72 with and without iron induction), biological triplicates were performed. Transcriptomic analyses monitor 236 transcriptional changes (with |log2 fold change| > 2 and *p*‐value < 0.001, Table S1) in response to 72 h of iron induction, corresponding to 10% of *T. kodakarensis* ' gene content (2306 coding genes identified from the KOD1 genome of 2,088,737 bp) (Fukui et al. 2005). No differential gene expression profiles were observed between the two T0 conditions (i.e., with or without iron induction), indicating that after a 5 min iron induction, no transcriptional change occurs. After 72 h without iron induction, 24 genes display expression changes, likely due to a prolonged stationary phase. These 24 genes are also identified in the condition after 72 h of iron induction. They are thus excluded from the analyses, as they do not show significant relevance to iron induction. Half of these genes encode hypothetical proteins, while the other half is related to central metabolism.

**FIGURE 5:**
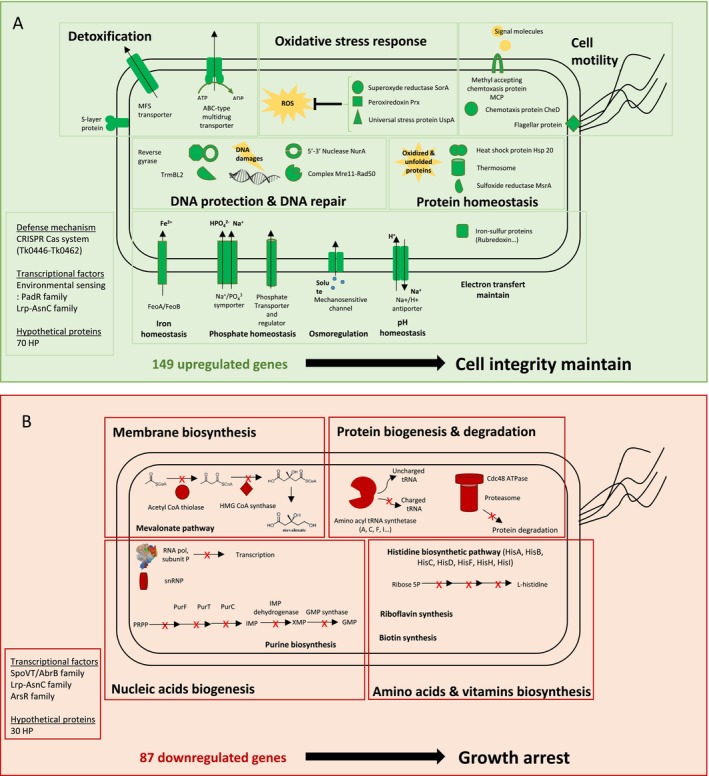
Summary of the upregulated (A) and downregulated (B) genes in the differential expression analysis of cells 72 h after 1 mM FeSO_4_ induction compared to cells that were grown in the absence of iron.

Among the 236 differentially expressed genes after 72 h of iron induction, 149 genes are upregulated (63%) (Table S1) and 87 are downregulated (Table S2). Approximately 40% (corresponding to 102/236 genes) of the regulated genes encode proteins with unknown functions and/or hypothetical proteins. This high proportion is consistent with previous transcriptomic studies conducted in Thermococcales, which reported that about 1/3 of the genes regulated in response to various stresses, such as exposure to metal, gamma irradiation or heat shock, also encode proteins of unknown function (Lagorce et al. 2012; Williams et al. 2007; Shockley et al. 2003). These findings highlight a recurring challenge in archaeal genomic studies: a substantial fraction of the stress response remains poorly understood at the molecular level. In this study, genes encoding proteins with known functions play a key role in general stress response mechanisms. They can be classified based on their involvement in similar biological processes, for example, such as detoxification, redox homeostasis, oxidative stress responses and DNA repair (Figure 5 and Table S1). Moreover, a coregulation of genes belonging to the same operon is observed; for instance, genes from Tk0244 to Tk0250 involved in the histidine biosynthesis pathway are all downregulated and genes from Tk0714 to Tk0716 implied in iron transport are upregulated. These findings suggest the biological relevance of this genome‐wide transcriptional analysis.

### 3.5 Upregulated Genes

Among the 149 upregulated genes, 79 encode proteins with known functions. Transcriptional responses 72 h post‐induction revealed that a significant proportion of the upregulated genes (29 out of 79, corresponding to 36%) encode transporters and permeases, with a log2 fold change ranging between 4 and 7. These are mainly related to drug/metabolite ABC transporters (Tk161–163; Tk692–694; Tk324–326; Tk1585–1588; Tk2053–2055; Tk2161–2162) and to the major facilitator superfamily (MFS, Tk0235) (Table S1). According to COG (cluster of orthologous genes) analysis, most of these transporters/permeases are classified as part of defence mechanisms (17/29), suggesting that they may help detoxify the cell, or that elevated iron levels interfere with the normal function of these membrane proteins, causing an imbalance in various ions. The remaining upregulated genes encoding transporters are involved in a range of other cellular processes, including ion transport and nutrient uptake, which help to maintain cellular homeostasis (Figure 5A).

*Thermococcus kodakarensis* harbours numerous genes involved in iron homeostasis, including those responsible for transport, regulation and storage, as this nutrient is essential for living cells. Its genome contains six operons associated with iron(III) transport (Tk0158–160; Tk570–572; Tk0706–0708; Tk0803–0804; Tk2018–2020; Tk2208–2209); and two operons dedicated to iron(II) transport (FeoB/FeoA Tk0714–0716 and Tk0957–0958). Additionally, two genes encoding ferric uptake regulation proteins (Tk1057, Tk1058), homologous to bacterial Fur proteins (Fukui et al. 2005), are present, although Fur homologue does not appear to play a role in the global regulatory response of cells to iron (Louvel et al. 2009). A third gene, TK0107, encodes a manganese‐dependent transcriptional regulator, a homologue of the bacterial DtxR protein, which may regulate the expression of iron acquisition proteins. These findings, previously reported in *T. kodakarensis*, have also been confirmed in *Pyrococcus furiosus* (Zhu et al. 2013), indicating this as a shared characteristic among Thermococcales. Finally, there are two genes encoding Ferritin‐like proteins (Tk1059, Tk1099), one of which is homologous to the Dps family protein. In this study, only the two operons encoding iron(II) transport proteins (FeoA/FeoB family proteins) are upregulated, indicating an increasing maintenance of iron(II) balance between the interior and exterior of the cell. Several genes involved in phosphate homeostasis are also upregulated, including the sodium‐dependent phosphate transporter (Tk2061). Inorganic phosphate is an essential element for nucleotide biosynthesis, energy supply and cellular signalling in living cells (Tsai et al. 2020). Phosphate becomes even more critical in iron‐rich anoxic environments, as it rapidly precipitates with iron(II), preferentially forming Fe(II) phosphates such as vivianite (Cosmidis et al. 2014). Due to the low solubility of phosphates in our mineralization medium containing iron(II), cells likely need to counterbalance this to maintain phosphate homeostasis. Other upregulated genes are involved in pH homeostasis (Tk2142), osmo‐regulation (Tk1940) and protein homeostasis (Tk2303, Tk1155), contributing to proper protein folding.

*T. kodakarensis* is well‐equipped to cope against oxygen stress (Fukui et al. 2005; Confalonieri and Sommer 2011). It encodes the superoxide reductase SOR instead of SOD (superoxide dismutase) in aerobic organisms, which detoxifies superoxide anions. The organisation of *sor* (Tk0525), alongside the rubredoxin (Tk0524) and ruberythrin (Tk0523) genes, is highly conserved among Thermococcales. In this transcriptomic analysis, the *sor* and rubredoxin gene are significantly upregulated (> 3), as well as a peroxiredoxin gene (Tk0537), which is also assumed to act on oxygen detoxification. Several studies have demonstrated that oxidative stress occurs in various cells exposed to iron through the production of reactive oxygen species (ROS) via the Fenton reaction. Moreover, *T. kodakarensis* harbours a distinct repair enzyme, a protein‐methionine sulfoxide reductase, Msr (Tk0819), which can reduce the methionine sulfoxide, considered as a reactive sulfur species (RSS). The mineralization conditions we conduct may indeed have generated oxidative stress, through the production of both ROS and RSS.

Many genes involved in DNA repair and chromosome structure are also upregulated with log2 fold changes exceeding 5. DNA repair genes are organized in operons and encode proteins conserved in thermophilic archaea. These include the 5′–3′ DNA repair nuclease NurA (Tk2210), the ATPase Rad50 (Tk2211), and the exonuclease Mre11 (Tk2212), all of which play crucial roles in archaeal recombination and repair mechanisms. In this transcriptomic analysis, the *trmBL2* (Tk 0471) and reverse gyrase (RG) (Tk0470) genes are upregulated. The DNA binding protein TrmBL2 is known to be involved in chromatin architecture (Maruyama et al. 2011), interacting with histones and the Alba protein, all of which are also conserved among Thermococcales. Recent studies have demonstrated that TrmBL2 can stabilise both single‐ and double‐strand DNA (Wierer et al. 2016). RG is the only enzyme present in all thermophilic species (Forterre 2002; Catchpole and Forterre 2019). Its role is to positively supercoil and compact DNA. It has been previously demonstrated that RG is more active after UV‐induced stress, which causes double‐strand breaks (DSBs) in Thermococcales DNA (Gorlas et al. 2019). As discussed previously, under the mineralization conditions of this study, the presence of soluble iron(II), the most toxic form of iron, could induce oxidative stress through the production of ROS and RSS, even in anoxic conditions. This oxidative stress could lead to DNA base modifications and mutations, single‐ and double‐strand breaks representing the most severe consequences. The proteins described above, whose genes are upregulated after 72 h of iron induction, are essential for repairing DNA (DSBs). Moreover, some genes involved in cell motility are overexpressed, suggesting a strategic adaptation employed by *T. kodakarensis* to move away from stressful conditions, probably microenvironments characterized by elevated soluble iron(II) levels or iron nanoparticles precipitation.

### 3.6 Downregulated Genes

Among the 87 genes downregulated, 57 encode proteins with known functions. The majority of downregulated genes are involved in the biosynthesis of macromolecules including nucleic acids, proteins, and lipids biosynthesis as well as in the biosynthesis of amino acids (specifically the histidine biosynthesis pathway) and vitamins (Table S2 and Figure 5B). Additionally, 15 out of 57 downregulated genes are related to transcription, nucleotide transport metabolism, translation, and ribosomal structure and biogenesis. Purines, which are essential for the biosynthesis of RNA and DNA, are usually synthesised through the purine biosynthesis pathway involving *pur* genes (Brown et al. 2011). In this study, all genes involved in the purine biosynthesis pathway are downregulated in *T. kodakarensis* post iron induction, with a |log2 fold change| > 3. As observed for *T. onnurineus*, *T. kodakarensis* may instead obtain purines through the histidine biosynthesis pathway (Lee et al. 2008). However, genes involved in the histidine biosynthesis pathway are similarly downregulated, suggesting that iron induction leads to growth decrease in *T. kodakarensis* .

To compose their cellular membranes, all Archaea use the isoprenoid‐based glycerol lipid ethers via the mevalonate pathway. *T. kodakarensis* genome contains the three genes involved in the acetyl‐CoA condensation to mevalonate (Liman et al. 2019): the acetoacetyl‐CoA thiolase (Tk0180), the HMG‐CoA synthase (Tk0181), and the HMG‐CoA reductase (Tk0914). Under iron induction, the two genes Tk0180 and Tk0181 are downregulated, with a |log2 fold change| > 4, indicating a decrease in membrane synthesis, consistent with the markers of decreasing growth rates previously proposed.

Overall, the iron exposure (after 72 h) induces a coordinated transcriptional response in *T. kodakarensis* , with strong regulation of stress‐related pathways and downregulation of biosynthetic processes, indicative of a metabolic shift toward survival.

## 4 Discussion

Extreme hyperthermophilic microorganisms have been isolated from hydrothermal vents (Takai et al. 2001; Huber et al. 1989; Blöchl et al. 1997), which are among the most biologically active regions of the deep ocean. These environments exhibit unique physical and chemical characteristics, including high hydrostatic pressure, elevated concentrations of dissolved metals (Holden and Adams 2003; Tivey 2007; Toner et al. 2016), and often high temperatures. Pyritization is a crucial process in these environments, participating in the formation of hydrothermal chimney structures. However, the precise mechanisms of pyrite formation, especially at temperatures below 150°C in the outer chimney regions, are still under discussion, with some research indicating that biological factors may play a role (Juniper et al. 1995; McCollom 2007). Recent research has specifically focused on understanding how hyperthermophilic archaea from the Thermococcales order, well represented in hydrothermal vents (Takai et al. 2001), contribute to the formation of iron sulfide minerals (Gorlas et al. 2022, 2018; Truong et al. 2023; Truong, Bernard, Baudin, et al. 2024; Mansor et al. 2025; Truong, Bernard, Gorlas, et al. 2024).

### 4.1 Iron Sulfide Biomineralization by Thermococcales

The production of pyrite spherules by Thermococcales has been well documented in previous studies using Thermococcales incubated at 85°C in their culture medium supplemented with aqueous solutions of Fe^2+^, most often sulfate salts at iron concentration of 5 mM (Gorlas et al. 2022, 2018; Truong et al. 2023; Truong, Bernard, Baudin, et al. 2024; Mansor et al. 2025; Truong, Bernard, Gorlas, et al. 2024). We simulated a specific and ecologically relevant scenario: one single 1 mM iron pulse applied to cells in late‐exponential phase, reflecting transient metal enrichments that can occur in natural hydrothermal systems (Tivey et al. 1990). The present work provides new insights into the characteristics of pyrite spherules formed in the presence of the hyperthermophilic archaeon *Thermococcus kodakarensis* under various mineralization conditions differing from those used in these previous studies. The diversity of conditions allowing the formation of pyrite spherules (Figure 1) reinforces the idea that this phenomenon might actually occur in hydrothermal vent environments (Gorlas et al. 2018). Indeed, a wide range of iron concentrations under various speciations has been reported close to black smokers inhabited by Thermococcales (Holden et al. 2012), and pyrite spherules sharing similarities to those produced by Thermococcales have been observed in the external part of the chimney wall in hydrothermal deep‐sea vent, at the Transatlantic Geotraverse (TAG) site, at 26°08′ N on the Mid‐Atlantic Ridge (MAR), making them a promising possible biosignature (Truong, Bernard, Baudin, et al. 2024).

Furthermore, previous studies have shown that pyrite spherules form only in the presence of Thermococcales cells, likely through the polysulfide pathways (Gorlas et al. 2022; Truong et al. 2023) from an iron monosulfide precursor FeS. However, the precise nature of the relationship between cells and pyrite spherules is not yet understood. It is indeed difficult to prove that some pyrite spherules are directly derived from individual cells. However, several observations made in the present study strongly suggest that this phenomenon actually occurs. A core‐shell structure (Figure S8) suggests mineral nucleation on cell surfaces similar to phosphate biomineralization described by Kish et al. (2016) In the case of Sulfolobales. And even more clearly, electron‐dense spherules have been observed to correspond to ancient, intact cells with retained their flagella (Figure S5D). The relative rarity with which these intermediate states are detected suggests that the transition from living cell to pyrite spherule is rapid and unsynchronized at the population level in those experimental conditions. Indeed, both mineralized and healthy cells coexist after 72 h of iron induction (Figure 4). One possible interpretation of these observations is that an abrupt and discontinuous cell death event exposes intracellular contents to extracellular dissolved iron‐rich medium, leading to iron nucleation at the interface of the cellular envelope, and allowing for mineralization from the outside in. In addition, an influx of Fe^2+^ linked to a disruption of intracellular iron homeostasis, possibly related to the interaction of the cell surface with iron‐bearing nanoparticles, could lead to nucleation of pyrite upon reaching a critical concentration necessary for reaction with intracellular sulfur, resulting in mineralization from the inside out. These suggestions are consistent with the proposal that pyrite spherule formation results from an induced biomineralization process rather than a genetically controlled one (Gorlas et al. 2022; Truong et al. 2023). The asynchronous transition from living cells to mineralized spherules implies that biomineralization is not a strictly regulated biological mechanism but a response to environmental conditions that drive iron–sulfur interactions.

The initial stage of iron precipitation occurs within the first hours following induction but it does not involve FeS_2_ pyrite formation. Instead, an iron sulfide matrix with a stoichiometry close to FeS precipitates. This matrix is quasi‐amorphous according to electron diffraction analysis and contains phosphorus. A part of the FeS detected at this stage is likely formed due to the presence of Na_2_S in the culture medium, as dissolved sulfides react abiotically with Fe^2+^. As shown in Figure 2, additional FeS is produced in the presence of cells. This biologically induced FeS precipitation is most likely the result of microbial reduction of elemental sulfur S(0), as shown in the case of 5 mM initial Fe^2+^ concentration by Truong et al. (2023). In the present study, one clearly observes by cryo‐electron microscopy, exclusively in biotic conditions, the formation of needle‐shaped FeS nanocrystals in close proximity to cells. These structures are suggestive of mackinawite (Picard et al. 2018; Zhou et al. 2017) although it was not possible to identify them by XRD (too small quantities) nor by SAED (complicated in cryo‐EM). Mackinawite is a metastable and highly fragile mineral, which may contribute to the difficulty in its detection and characterisation in our samples. Interestingly, these needle‐shaped FeS nanocrystals are never observed in abiotic conditions. This supports the idea that microbial processes influence the morphology of FeS, as previously reported (Mansor et al. 2025; Picard et al. 2018; Zhou et al. 2017). The formation of well‐crystallized needles of mackinawite has been linked to the presence of microorganisms capable of reducing sulfur compounds (Picard et al. 2018; Zhou et al. 2017). Similarly, we suggest that the biological activity of Thermococcales promotes the formation of well‐crystallized FeS mackinawite needles.

A second stage of iron precipitation occurs after 72 h, during which FeS produced in the initial stage reacts with reactive sulfur provided from lysed cells and free sulfur vesicles (Gorlas et al. 2015), yielding FeS_2_ pyrite. Lysis of part of the cells, releasing reactive sulfur necessary to the formation of pyrite spherules, could be caused either by oxidative and physical stresses from iron ions and/or nanoparticles, or as a natural consequence of the transition into stationary phase. We suggest that the cellular envelope acts as a scaffold, allowing pyrite to mineralize into a smooth sphere, in contrast to the pyrite spherules produced under abiotic conditions supplemented with colloidal sulfur (Truong, Bernard, Baudin, et al. 2024). In such biotic conditions, the reactive sulfur facilitates the production of pyrite spherules that have a distinct smooth exterior (Truong et al. 2023), due to the size of each nanopyrite domain (measuring 10–15 nm in diameter) (Figure S1). The organic matrix surrounding pyrite nanocrystals prevents their further growth by acting as a structural network, while also serving as a nucleation interface between reactive sulfur and reduced iron. In Thermococcales, the cellular envelope is a S‐layer, a pseudocrystalline array of (glyco)proteins (Albers and Meyer 2011; Rodrigues‐Oliveira et al. 2017), which can function as a template for mineralization (Kish et al. 2016; Sleytr et al. 2014). Owing to its protein arrangement, the S‐layer is highly porous and able to entrapping metallic ions (Velasquez and Dussan 2009). In this study, we propose that the Thermococcales S‐layer promotes the formation of biogenic pyrite by facilitating the specific interactions between iron and sulfur, thereby ensuring high control over nanocrystal size and stability. To fully understand how the S‐layer organises these interactions and how microbial cells control pyrite biomineralization, a detailed analysis of the S‐layer structure and of its composition must be carried out in the future.

The necessity of intracellular reactive sulfur for pyrite formation is confirmed by glutaraldehyde fixation experiments, in which elimination of cellular contents resulting from fixation and washing resulted in no pyrite formation. This suggests that the cellular envelope alone is not sufficient for pyrite nucleation, a conclusion also supported by Truong, Bernard, Gorlas, et al. (2024) through experiments using archaeal envelope fractions. However, even fixation at low temperatures (with samples kept on ice), which preserved most intracellular contents, did not result in pyrite formation. This indicates that access of iron to intracellular content may have been inhibited by the fixation process. An iron–sulfur interface is necessary for pyrite formation and could occur in natural settings through cell lysis, secretion of sulfur vesicles (Gorlas et al. 2015), or possibly the exposure of reactive sulfur at the surface of cells. The vesicles contain reactive sulfur (Gorlas et al. 2022, 2015), which may react with reduced iron upon contact. This interaction might represent the initial stage of heart‐shell structure (Figure S8) formation process and could also serve as an iron‐trapping mechanism. At lower iron concentrations, these contact points could immobilise iron similarly to whole cells, which would only achieve complete mineralization at higher iron concentrations. Additional experiments across a wide range of low iron concentrations would be necessary to further examine this hypothesis.

In this study, the low‐temperature and glutaraldehyde‐fixation experiments provide new insights on the relative contribution of abiotic and biotic factors in pyrite spherule formation. These results are fully consistent with our previous works, which had already suggested that cell surfaces and biomass (living or dead), rather than metabolically active cells, control pyrite nucleation and growth (Truong, Bernard, Baudin, et al. 2024; Truong, Bernard, Gorlas, et al. 2024). The glutaraldehyde experiments refine this interpretation by showing that chemical crosslinking of surface proteins prevents pyrite formation, indicating that chemically intact and native cell surfaces are required to provide nucleation sites for pyrite formation. Moreover, pyrite spherules with the same morphology and size as those formed at 85°C can also form at 25°C–45°C, in lower abundance, demonstrating that pyrite formation is thermally activated but does not require active metabolism.

### 4.2 Rates of Formation of FeS and FeS_2_

Regarding timing, the formation of iron sulfides in the presence of Thermococcales can be divided into three main steps, as illustrated in Figure 2:

1. Immediately after the addition of Fe^2+^ to the medium, a rapid and massive precipitation occurs, mainly of FeS (see previous section), regardless of whether Thermococcales cells are present or not. This initial precipitation instantly reduces the concentration of dissolved iron from 1 mmol/L to approximately 140 μmol/L.
2. Precipitation of Fe^2+^ continues throughout the experiment, albeit slowing significantly, with very different kinetics in the absence or presence of cells.
3. A gradual transformation of FeS into FeS_2_ is observed exclusively in the presence of cells, beginning after a few tens of hours. This transformation leads to the almost complete resorption of FeS after about 100 h, with reoccurrence of FeS at very long times (> 200 h), suggesting a dynamic cycling process.

Following the initial, instantaneous formation of FeS, corresponding to the abiotic reaction of Fe^2+^ with HS^−^ (whose presence is due to Na_2_S addition prior to iron induction), further evolution of iron precipitation is very different in the absence and presence of cells. Under abiotic conditions, slow iron precipitation occurs with an average rate of approximately 0.01 mmol/L/day (as estimated from Figure 2). A closer examination of Figure 2 suggests that this average rate is around 0.03 mmol/L/day during the first 24 h, and then below 0.01 mmol/L/day. This is consistent with a faster rate when Fe^2+^ and HS^−^ concentrations are higher and with long characteristic time (typically exceeding 10 days) for reaching equilibrium (calculated to be few μmol/L of Fe^2+^ in equilibrium with amorphous FeS at 85°C and pH 7). In stark contrast, the presence of cells leads to higher FeS formation rates during the first 24 h, exceeding 0.1 mmol/L/day. These elevated rates are most likely driven by microbial reduction of zero valent sulfur, which produces a continuous supply of HS^−^. Beyond 24 h, the kinetics of FeS formation in the presence of cells is much more difficult to interpret. While FeS continues to be produced by reduction of zero‐valent sulfur which is always in excess, it is also simultaneously transformed into FeS_2_ pyrite. As discussed in the previous section, most of the FeS is converted to pyrite through interactions with sulfur vesicles over a period of around 48 h; we deduce a pyrite formation rate of around 0.5 mmol/L/day. This rate is slightly faster than that proposed by Mansor et al. for the bioreduction of zero‐valent sulfur at 85°C (see their figure 3, Mansor et al. 2025), but remains very consistent if one takes into account the substantial uncertainties on the effective interaction times between FeS and cells. Finally, at extended time scales, beyond 8 days, the activity of zero‐valent sulfur decreases, and therefore also the production of sulfides and polysulfides, and the slow precipitation of FeS continues from Fe^2+^ and HS^−^, asymptotically down to few μM of Fe^2+^.

### 4.3 Temperature Effect

In the present study, it was shown that pyrite spherules formed in experiments conducted at temperatures of 45°C and 25°C (Figure S3), that is, below the reported optimal growth temperature range of *T. kodakarensis* growth. However, the number of spherules formed at these lower temperatures was significantly reduced compared to those formed at 85°C. Although it is impossible to determine quantitatively the number of pyrite spherules produced from observations on electron microscopy grids only, a relative order of magnitude of the effect of temperature on this production can be estimated. At 85°C, the pyrite production thus calculated is of 0.4 mmol/L/day that is remarkably consistent with the value obtained from measurements of iron uptake from solutions (0.5 mmol/L/day, see previous paragraph). Based on statistical analysis of over 100 pyrite spherules in the squares of electron microscopy grids (see a selected example in Figure S3), the ratios of pyrite spherules production at 85°C relative to 45°C and 25°C are approximately 10:1 and 100:1, respectively. This allows to roughly estimate an activation energy for this process in the range of 70–130 kJ/mol. Such a value is more consistent with reaction processes, including enzymatic ones (such as metabolic zero‐valent sulfur reduction), or solid‐state diffusion, rather than with diffusion in aqueous solutions or in gel‐like environments (such as in sulfur vesicles). In any case, whether or not the cells are metabolically active at such sub‐optimal temperatures, this non‐zero low‐temperature production of pyrite spherules suggests the biogeochemical importance of dormant or of very weakly active cells at temperatures far below their metabolic optimum (Bradley 2025). Additional further investigations could help address this question through dosages of sulfur species in such low‐temperature experiments.

### 4.4 Iron Concentration and Speciation

In this study, we demonstrated that both iron concentration and speciation have a noticeable impact on pyrite formation. While lowering iron sulfate concentration from 5 to 1 mM did not lead to changes in first pyrite detection times (i.e., 72 h after induction) (Gorlas et al. 2022; Truong et al. 2023), a further reduction to 0.1 mM significantly delayed this first detection by several days (Figure S2). This observation supports the hypothesis (see previous paragraph on rates of formation) that pyrite forms from a pre‐existing FeS phase, whose available surface area is much smaller at 0.1 mM initial Fe^2+^ than at higher iron concentrations. We have also shown that switching the Fe^2+^ source from iron sulfate to iron chloride accelerated pyrite nucleation, with earlier detection at 24 h after induction (Figure S2). Sulfate ions have a greater affinity for Fe^2+^ than chloride ions, which could lead to an acceleration of FeS formation kinetics in the latter case, and hence a higher pyrite formation rate in presence of chloride ligands than with sulfate ligands. Systematic measurements of iron in these two conditions would help clarify the origin of this phenomenon.

### 4.5 Cellular Adaptation to the Hydrothermal Environment

The transcriptomic data collectively suggest that mineralization conditions impact and stress *T. kodakarensis* living cells, long after iron induction (after 72 h). This stress triggers a broad and global cellular response, characterized by the upregulation of genes involved in oxidative stress defense, DNA repair, and efflux pump systems, alongside the downregulation of biosynthetic pathways, including those for nucleic acids, proteins, and lipids (Figure 5). The persistence of this widespread response highlights the ongoing challenge for *T. kodakarensis* cells to manage and adapt to the high levels of soluble iron. These transcriptomic patterns are consistent with iron quantification measurements, which revealed a prolonged exposure to soluble iron (up to 25 μM Fe^2+^ after 72 h). This suggests that the cells are still actively engaged in counteracting the toxic effects of the iron, potentially at the cost of cellular growth and biosynthesis. This interpretation contrasts with observations in *T. gammatolerans* exposed to cadmium (Lagorce et al. 2012), where the stress response was restricted to metal detoxification, without significant downregulation of anabolic functions or induction of DNA repair systems, suggesting a different degree of adaptation. This difference may be explained by the duration of metal exposure: our study focuses on a long‐term (72 h) response to iron, indicating a strong cellular adaptation signature characterized by a redirection of energy from anabolic processes to detoxification and genome maintenance, whereas Lagorce et al. investigated early transcriptomic responses within the first 2 h after cadmium exposure, reflecting more immediate metal detoxification mechanisms without evidence of a general stress response (Lagorce et al. 2012). Indeed, additional transcriptomic analyses at earlier time points would be essential to distinguish primary iron‐sensing responses from secondary stress adaptations in our study.

Up‐regulation of genes involved in oxidative stress response and sulfoxide detoxification suggests that the mineralisation conditions in our experiments may have produced reactive oxygen species (ROS) or reactive sulfur species (RSS) or both. A possible explanation is that trace amounts of O_2_ are introduced during inoculation into the mineralization medium, which could then evolve into small amounts of ROS. Another, more likely scenario, though requiring further investigation, is that ROS could be generated even under anoxic conditions through interactions between Fe^2+^, H_2_O and/or organic molecules. Indeed Gorlas et al. (2018, 2022) and Truong, Bernard, Baudin, et al. (2024) have demonstrated the significant formation of Fe^3+^‐bearing phases under such anoxic conditions (Gorlas et al. 2022, 2018; Truong et al. 2023), which could indicate the presence of ROS. Once those potential ROS are formed, they could also lead to the formation of RSS in those sulfur‐rich media. Alternatively, polysulfides, which are almost certainly generated in this system (Truong, Bernard, Baudin, et al. 2024), might contribute to the upregulation of genes coding for methionine sulfoxide reductase (Msr).

The toxicity of iron thus manifests through both oxidative damage and physical damage to cells, the latter induced by FeS precipitation (Figure S6). Pyrite mineralization, whose detection occurs 72 h after iron addition, can in fact be interpreted as a form of iron and polysulfide detoxification that leads to the death of some cells but makes the environment more habitable for others. In this model, the classical mechanisms of detoxification of iron, ROS and/or RSS having been exceeded, a cell subpopulation favours the precipitation of cell‐damaging FeS and subsequently the formation of FeS_2_ pyrite, which consumes reactive sulfur species. This enables the surviving cell subpopulation to activate the classical mechanisms of iron, ROS or RSS detoxification pathways that we have highlighted in the transcriptomic study. Similar cell mechanisms have been described in other microorganisms such as *Desulfovibrio vulgaris* . For instance, the Cr(VI) immobilization through its reduction to Cr(III) (Cr_6_/Cr_3_) reduces toxicity (Goulhen et al. 2006). The accumulation of precipitated Cr(III) occurs on the cell surface, leading to the death of a part of the population while simultaneously reducing the redox potential of the culture medium to a tolerable level for the bacteria. Additionally, during the early log phase, Cr(III) phosphate may form on the cell and within the membrane. A comparable process was observed during the mineralization process of Thermococcales, with initial formation of Fe(III) phosphate on the cell (Gorlas et al. 2022, 2018; Truong et al. 2023). The detoxification thus seems to involve two distinct task‐specific groups within the population. Indeed, living cells are still observed in microscopy alongside mineralized cells (Figure 4). Flow cytometry measurements indicate a heterogeneous population emerging during the transition from early to late stages (96 h). While a part of the cell population remains unchanged, another part of the population is probably dead, displaying increased granularity and loss of SYBR signal. This loss in SYBR signal probably results from the mineralizing FeS or pyrite crusts, which prevent the fluorophore's ability to penetrate the cells. The former population are cells that survive the induction, whereas the latter represents the group of mineralizing cells.

This population heterogeneity is likely driven by stochastic effects, related to the Brownian motion of FeS nanoparticles in the medium. We suggest that Thermococcales cells in direct contact with the bulk of dissolved iron induce the precipitation of iron, thereby lowering the iron concentration in the surrounding environment. These biomineralizing cells may thus act as sacrificial cells and may serve a secondary role in nutrient storage. The remaining cells deal with the residual dissolved iron through a variety of molecular mechanisms, including the overexpression of genes involved in oxidative stress defense, DNA repair, and efflux pump systems. At the same time, they adapt to reduced resource availability by downregulating pathways related to biosynthesis, including those for nucleic acids, proteins, and lipids. Population heterogeneity, especially when task‐specific, is a common characteristic of biofilms and multicellular organisms. This population dynamic can be compared to the impact of bactericidal drugs on microbial colonies. Research has demonstrated that sub‐MIC (minimum inhibitory concentration) levels of drugs influence population dynamics, causing antibiotic‐resistant bacteria to variably survive or perish, even when they originated from genetically identical cells cultured under homogeneous antibiotic conditions (Coates et al. 2018). Such stochastic variation in population behaviour could be explained by several factors: an intrinsic variability in gene expression (i.e., expression of antibiotic resistance genes); a cellular persistence, where dormant cells are refractory to antibiotics; and the non‐uniform distribution of antibiotics within the medium, which could be similar to the stochastic distribution of colloidal FeS nanoparticles in the medium, during the mineralization process of Thermococcales.

## 5 Conclusion

This study provides new insights into the biogenic formation of iron sulfides by Thermococcales, demonstrating a significant microbial influence on the precipitation of well‐crystallized FeS and its transformation into FeS_2_ pyrite. Our findings highlight the critical role of intracellular reactive sulfur and the structural features of the S‐layer in governing pyrite nucleation and growth in laboratory conditions. Notably, the pyrite formation occurs even at sub‐optimal growth temperatures and under low iron availability, suggesting that Thermococcales may contribute to iron and sulfur cycling along hydrothermal chimney gradients, beyond their optimal thermal niche. Although our study focuses on a scenario that is likely to occur in hydrothermal ecosystems, a single iron pulse applied to cells in late exponential phase; other exposure scenarios exist such as continuous iron supply and/or repeated iron pulses and could trigger distinct regulatory responses. Future studies using high‐temperature fed‐batch bioreactors will allow us to explore these alternative conditions, enhancing the biological relevance of our findings. Overall, this work contributes to a better understanding of microbially mediated mineralization processes and reinforces the value of Thermococcales as model organisms for studying biogeochemical transformations in extreme environments.

## Author Contributions

**A. Gorlas:** conceptualization, investigation, funding acquisition, writing – original draft, methodology, validation, writing – review and editing, visualization, project administration, data curation, supervision, resources. **R. Coudray:** writing – review and editing, investigation. **C. Toffano‐Nioche:** software, formal analysis, methodology, writing – review and editing. **T. Mariotte:** conceptualization, investigation, writing – original draft, writing – review and editing, methodology, validation, visualization, software, resources. **F. Guyot:** supervision, writing – review and editing, writing – original draft, investigation, conceptualization, methodology, validation.

## Funding

This work was supported by Agence Nationale de la Recherche, ANR‐20‐CE02‐0001‐01.

## Conflicts of Interest

The authors declare no conflicts of interest.

## Supporting information


**Figure S1:** Pyrite spherules (A) and their sections (B, C), in *Thermococcus kodakarensis* culture 72 h after induction (FeSO_4_ 1 mM) under scanning electron microscope (SEM) (A–C), before (A) and after (B, C) milling performed by focused ion beam (FIB). X‐ray crystal diffraction pattern (D) from (C) with indexation showing the attribution of the pattern to pyrite (FeS_2_).
**Figure S2:** Observation of the first iron sulfide spherules produced in each condition, under 1 mM of FeSO_4_ after 72 h of mineralization (A), 1 mM of FeCl_2_ after 24 h of mineralization (B), 0.1 mM of FeSO_4_ (C) and 0.1 mM of FeCl_2_ both after 20 days of mineralization (D).
**Figure S3:** Scanning electron microscopy images of culture samples from BM experiments from 20 days after glutaraldehyde fixation and induction with 1 mM FeSO_4_ (A), and 20 days after induction at different temperatures: 25°C (B), 45°C (C), 85°C (D).
**Figure S4:** Penicillin flasks of *T. kodakarensis* culture before and after iron induction (A) along an optical microscopy image of cells after induction (B), showing the dark precipitates forming in the medium.
**Figure S5:** Transmission electron microscopy images (A–F) of iron‐induced culture medium without cells (B) and *Thermococcus kodakarensis* cells (A, C–F) before induction with 1 mM FeSO_4_ (A), and 1 h (C), 96 h (D), 144 h (E) and 192 h (F) after induction.
**Figure S6:** *Thermococcus kodakarensis* cells (FeSO_4_ 1 mM) in a mineral matrix: experiencing damage from penetrating FeS and leaking intracellular content (A–D: MET; E–H: cryo‐MET).
**Figure S7:** *Thermococcus kodakarensis* cells (FeSO_4_ 0.1 mM) and numerous budding vesicles, including a sulfur vesicle, and surrounding FeS mineral matrix (cryo‐MET).
**Figure S8:** Cryo‐EM image of *Thermococcus kodakarensis* cell, 1 h after iron induction, with a close‐up of a heart‐shell structure suspected to be a nucleation point for pyrite.
**Table S1:** Table of upregulated genes in differential expression analysis.
**Table S2:** Table of downregulated genes in differential expression analysis.

## Data Availability

The data and materials that support the findings of this study are available in the article or in Appendix S1. The raw transcriptomic data are available have been deposited in the European Nucleotide Archive (ENA) at EMBL‐EBI under accession number PRJEB105195.
