# Supplementary material for: Iron Sulfides Produced by Thermococcales: An Iron Detoxification Mechanism
Source: Environ Microbiol. 2026 Jan 22;28(1):e70242. doi: 10.1111/1462-2920.70242 (PMC12827226; doi:10.1111/1462-2920.70242)
Supplement: Supplementary file 1 — Figure S1: Pyrite spherules (A) and their sections (B, C), in Thermococcus kodakarensis culture 72 h after induction (FeSO4 1 mM) under scanning electron microscope (SEM) (A–C), before (A) and after (B, C) milling performed by focused ion beam (FIB). X‐ray crystal diffraction pattern (D) from (C) with indexation showing the attribution of the pattern to pyrite (FeS2). Figure S2: Observation of the first iron sulfide spherules produced in each condition, under 1 mM of FeSO4 after 72 h of mineralization (A), 1 mM of FeCl2 after 24 h of mineralization (B), 0.1 mM of FeSO4 (C) and 0.1 mM of FeCl2 both after 20 days of mineralization (D). Figure S3: Scanning electron microscopy images of culture samples from BM experiments from 20 days after glutaraldehyde fixation and induction with 1 mM FeSO4 (A), and 20 days after induction at different temperatures: 25°C (B), 45°C (C), 85°C (D). Figure S4: Penicillin flasks of T. kodakarensis culture before and after iron induction (A) along an optical microscopy image of cells after induction (B), showing the dark precipitates forming in the medium. Figure S5: Transmission electron microscopy images (A–F) of iron‐induced culture medium without cells (B) and Thermococcus kodakarensis cells (A, C–F) before induction with 1 mM FeSO4 (A), and 1 h (C), 96 h (D), 144 h (E) and 192 h (F) after induction. Figure S6: Thermococcus kodakarensis cells (FeSO4 1 mM) in a mineral matrix: experiencing damage from penetrating FeS and leaking intracellular content (A–D: MET; E–H: cryo‐MET). Figure S7: Thermococcus kodakarensis cells (FeSO4 0.1 mM) and numerous budding vesicles, including a sulfur vesicle, and surrounding FeS mineral matrix (cryo‐MET). Figure S8: Cryo‐EM image of Thermococcus kodakarensis cell, 1 h after iron induction, with a close‐up of a heart‐shell structure suspected to be a nucleation point for pyrite. Table S1: Table of upregulated genes in differential expression analysis. Table S2: Table of downregulated genes in differential [file EMI-28-e70242-s001.docx]

# Supplementary material


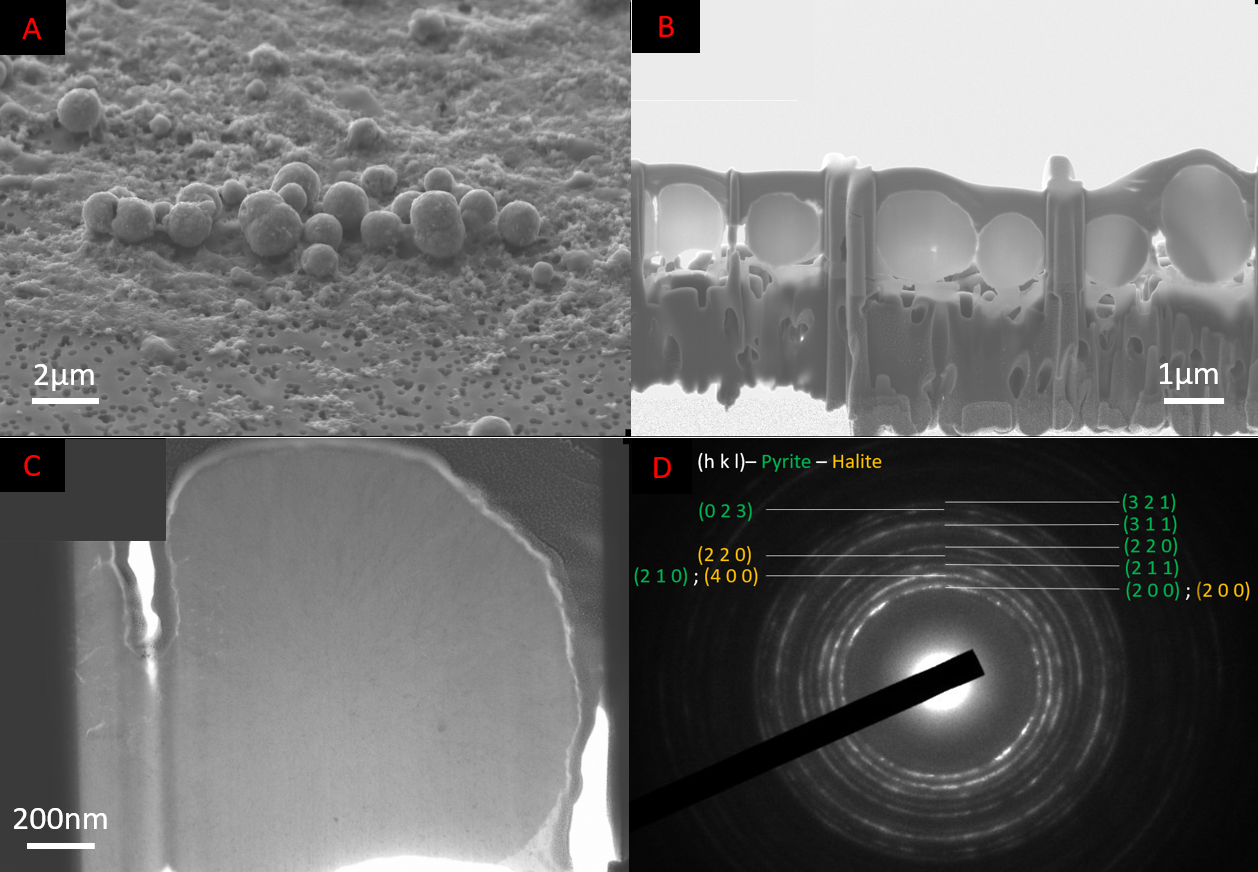


Fig S1: Pyrite spherules (A) and their sections (B,C), in Thermococcus kodakarensis culture 72 hours after induction (FeSO_4_ 1mM) under Scanning electron microscope (SEM) (A,B,C), before (A) and after (B,C) milling performed by Focused Ion Beam (FIB). X-Ray crystal Diffraction pattern (D) from (C) with indexation showing the attribution of the pattern to Pyrite (FeS_2_).


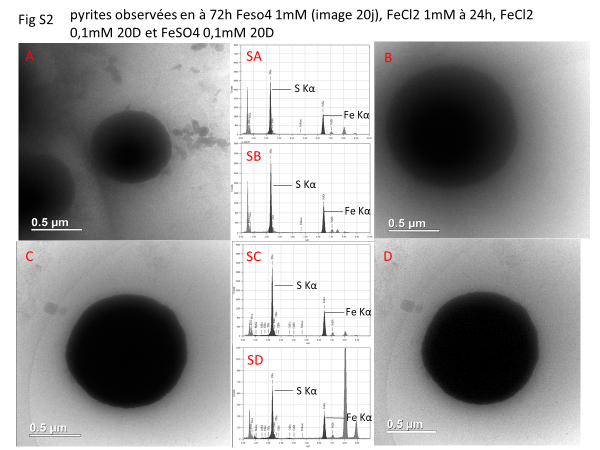


Fig S2: Observation of the first iron sulfide spherules produced in each condition, under 1mM of FeSO_4_ after 72 hours of mineralization (A), 1mM of FeCl_2_ after 24 hours of mineralization (B), 0.1 mM of FeSO_4_ (C) and 0.1 mM of FeCl_2_ both after 20 days of mineralization (D).


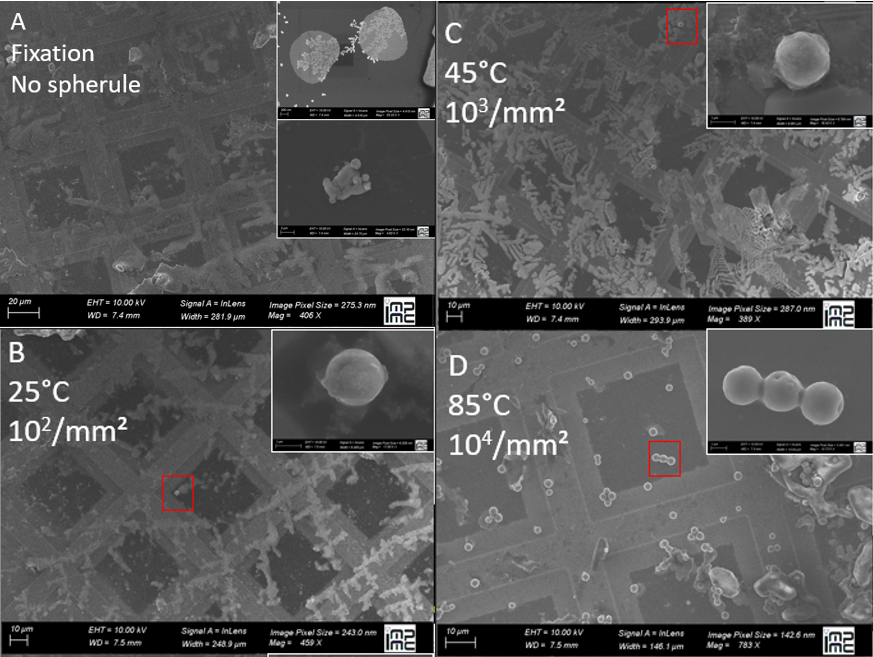


Fig S3: Scanning electron microscopy images of culture samples from BM experiments from 20 days after glutaraldehyde fixation and induction with 1mM FeSO_4_ (A), and 20 days after induction at different temperatures: 25°C (B), 45°C (C), 85°C (D).


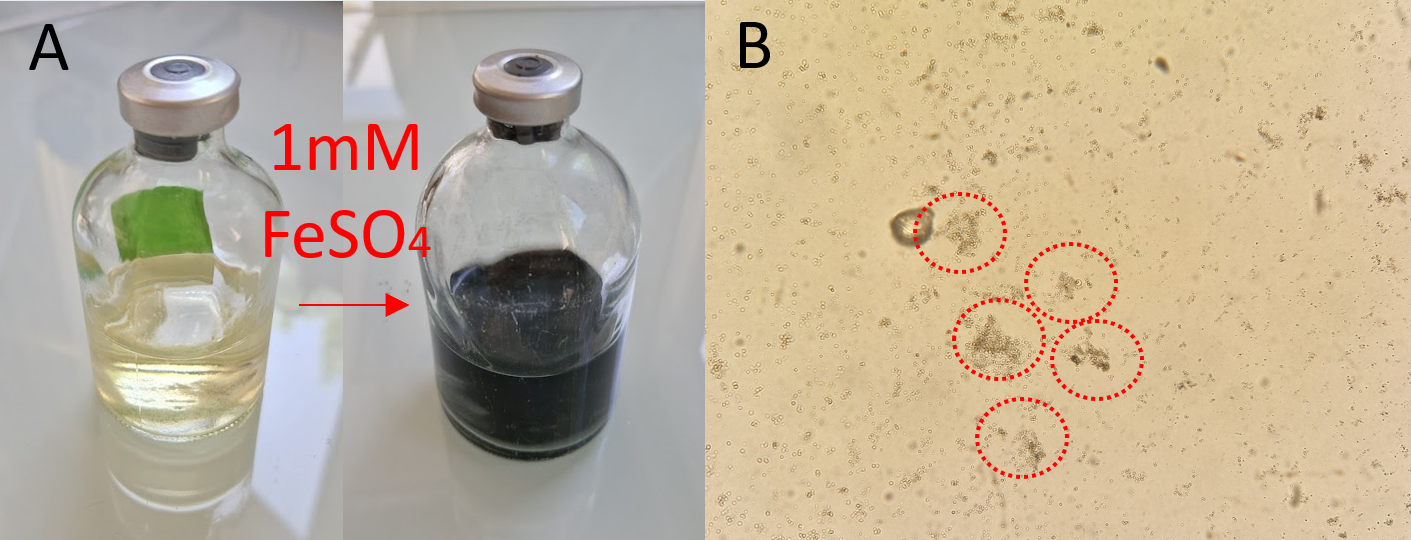


Fig S4: Penicillin flasks of *T. kodakarensis* culture before and after iron induction (A) along an optical microscopy image of cells after induction (B), showing the dark precipitates forming in the medium.


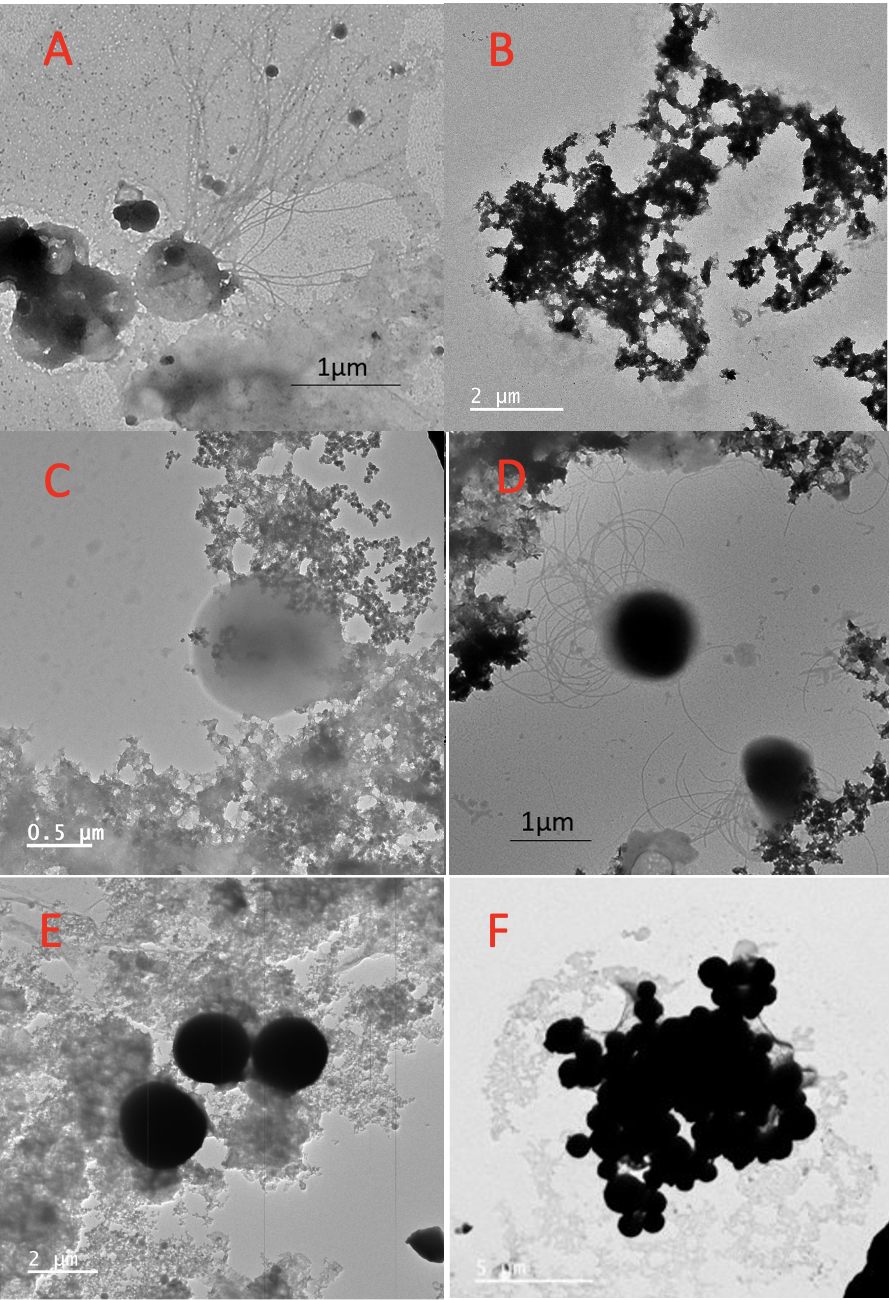
Fig S5: Transmission electron microscopy images (A-F) of iron-induced culture medium without cells (B) and *Thermococcus kodakarensis* cells (A, C-F) before induction with 1mM FeSO_4_ (A), and 1h (C), 96h (D), 144h (E) and 192h (F) after induction.


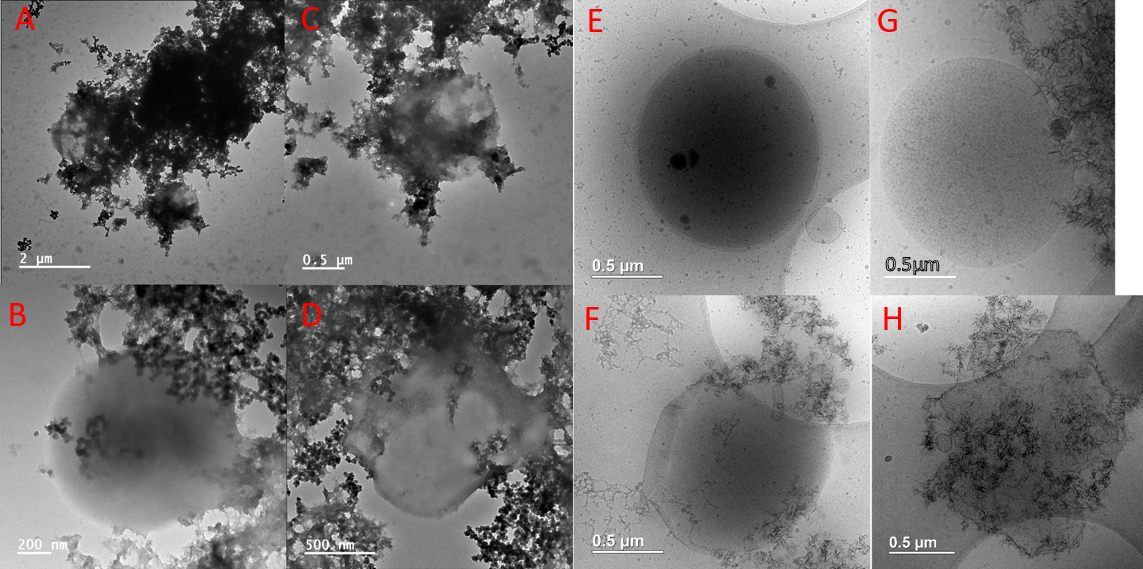


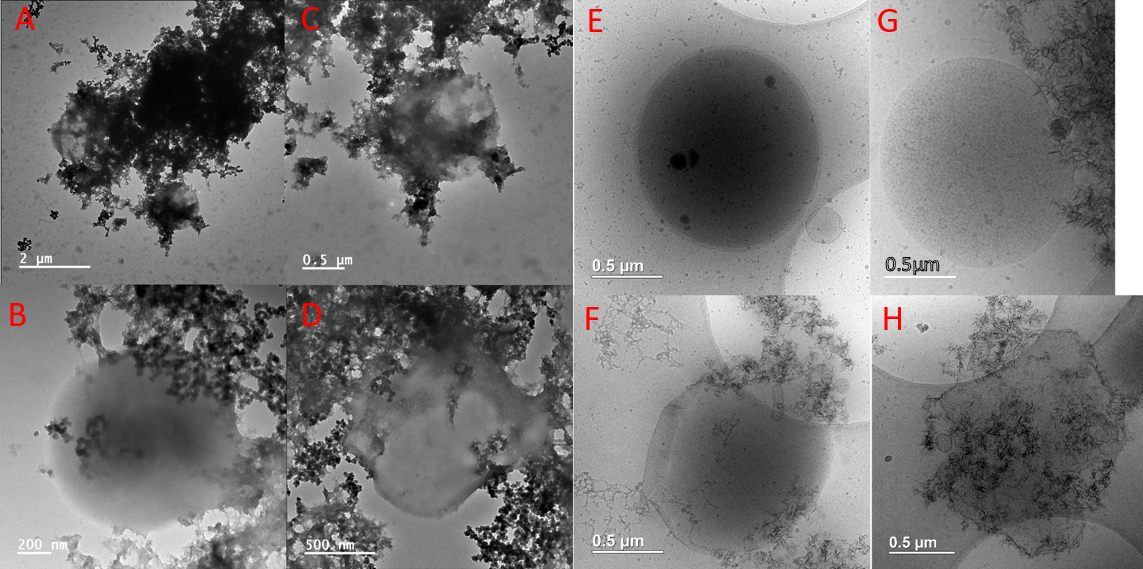


Fig S6: Thermococcus kodakarensis cells (FeSO_4_ 1mM) in a mineral matrix: experiencing damage from penetrating FeS and leaking intracellular content (A-D: MET; E-H: Cryo-MET).


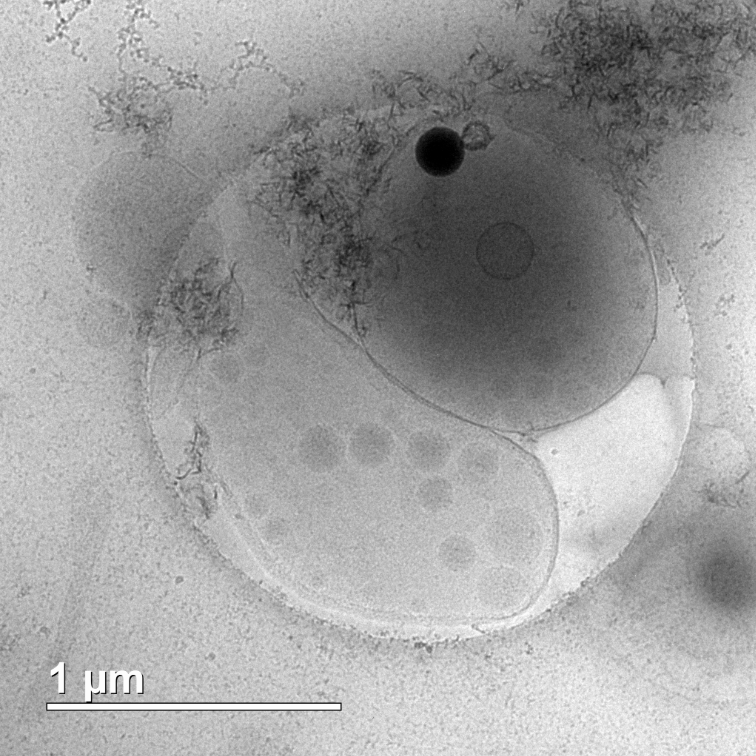


Fig S7: Thermococcus kodakarensis cells (FeSO_4_ 0.1 mM) and numerous budding vesicles, including a sulfur vesicle, and surrounding FeS mineral matrix. (Cryo-MET)


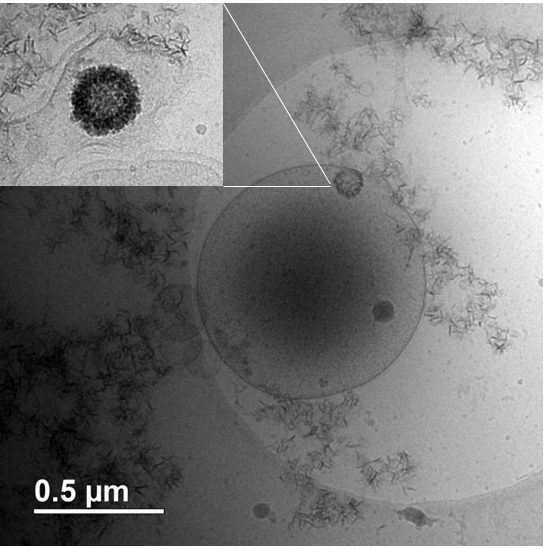


Fig S8: Cryo-EM image of *Thermococcus kodakarensis* cell, 1h after iron induction, with a close-up of a heart-shell structure suspected to be a nucleation point for pyrite.


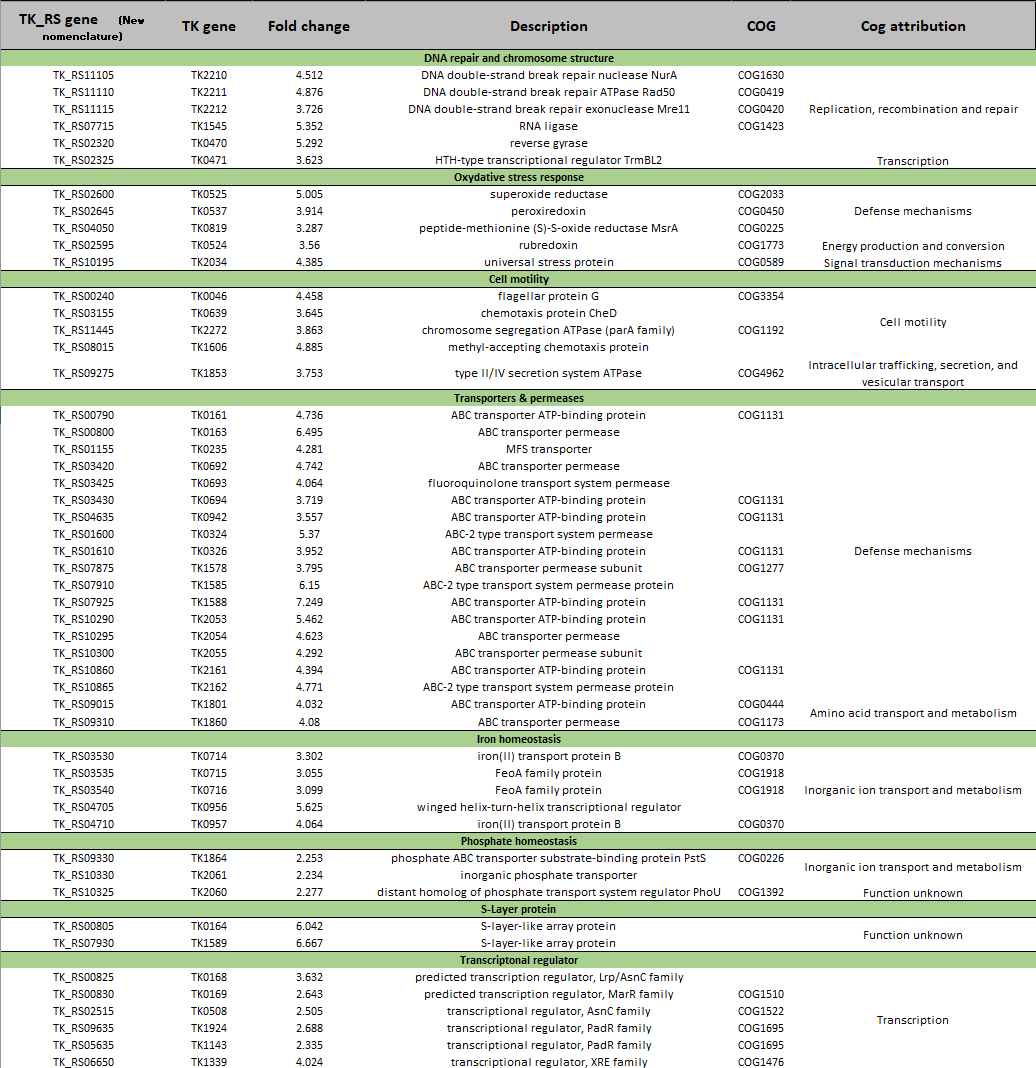

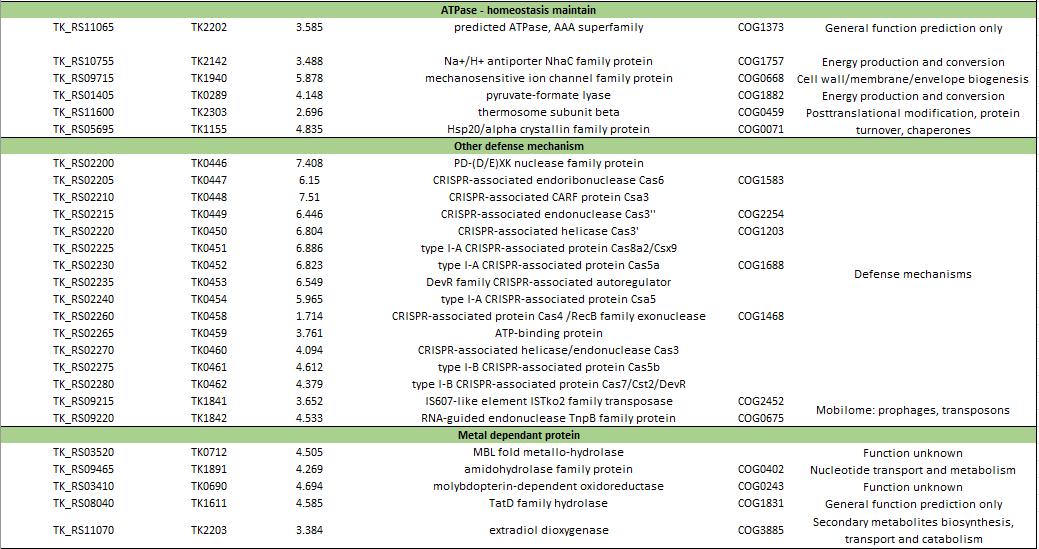


Table S1 : Table of upregulated genes in differential expression analysis.


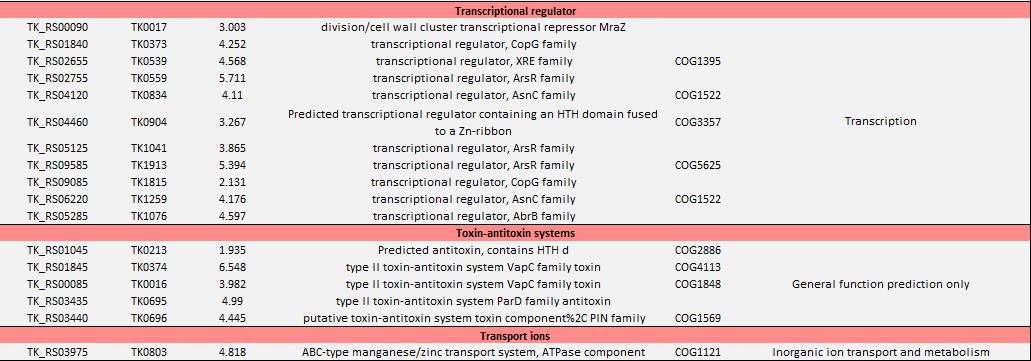

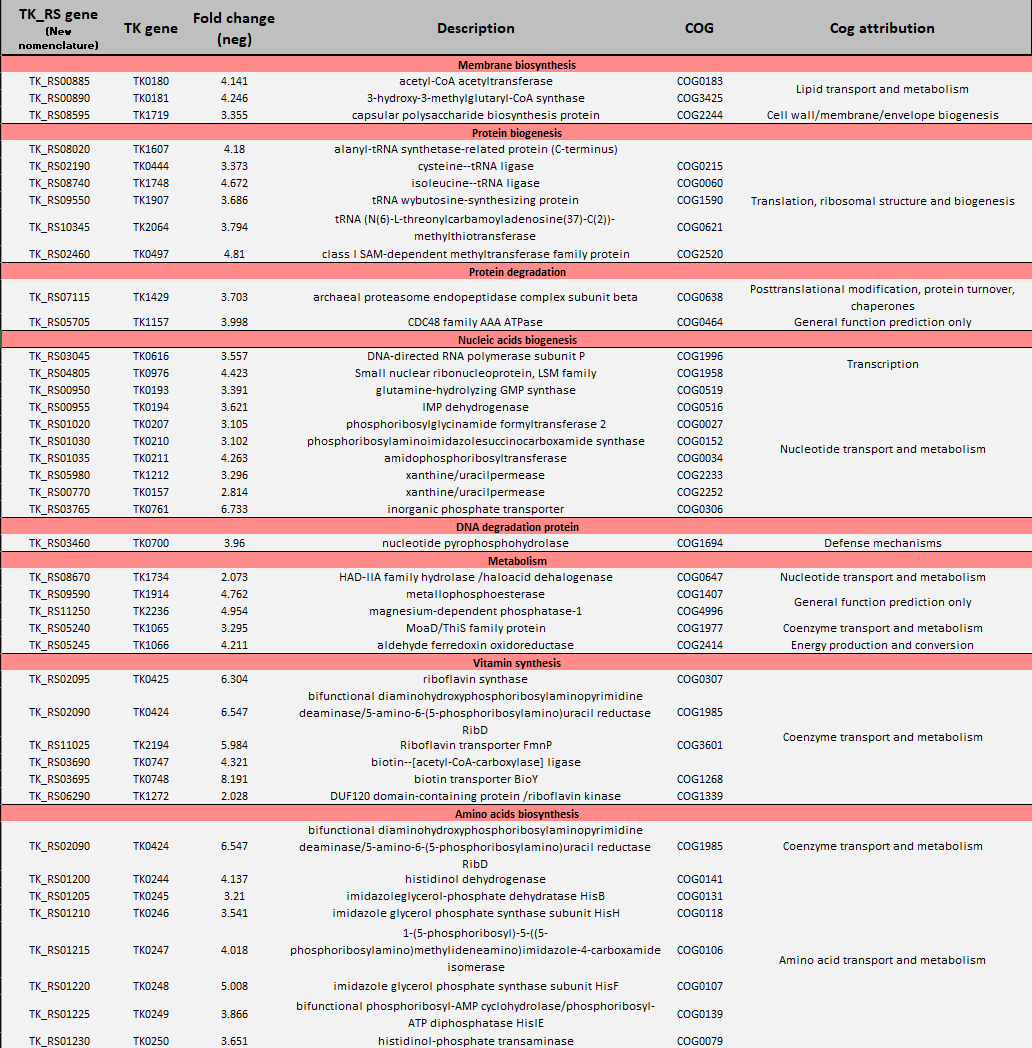


Table S2 : Table of downregulated genes in differential expression analysis
